# Asymmetric gating of a homopentameric ion channel GLIC revealed by cryo-EM

**DOI:** 10.1073/pnas.2512811122

**Published:** 2025-10-23

**Authors:** Zhuowen Li, Nikhil Bharambe, Kashmiri Manishrao Lande, Bjarne Feddersen, Asha Manikkoth Balakrishna, Philip C. Biggin, Giriraj Sahu, Sandip Basak

**Affiliations:** ^a^School of Biological Sciences, Nanyang Technological University, Singapore 637551, Singapore; ^b^Molecular Biophysics Unit, Indian Institute of Science, Bangalore 560012, India; ^c^Structural Bioinformatics and Computational Biochemistry, Department of Biochemistry, University of Oxford, Oxford OX1 3QU, United Kingdom; ^d^Nanyang Technological University (NTU) Institute of Structural Biology, Nanyang Technological University, Singapore 639798, Singapore

**Keywords:** ligand-gated Ion channel, cryo-EM, MD simulations, nanodisc, patch-clamp

## Abstract

Pentameric ligand-gated ion channels (pLGICs) are vital neurotransmitter receptors that are key therapeutic targets for neurological disorders. Although the high-resolution structures of these channels have been elucidated, capturing their dynamic conformational transitions remains challenging due to the transient nature of intermediate states. In this study, we investigated a prokaryotic proton-gated pLGIC, GLIC. In our cryo-EM data at pH 4.0, we identified and segregated asymmetric particles, which we precisely aligned to resolve high-resolution structures of several previously unresolved asymmetric intermediate states, in addition to symmetric closed and open states. Detailed structural analysis revealed systematic conformational changes at individual subunits driving the channel opening. Molecular dynamics simulations were used to assign the functional states. We further examined the roles of the F116 and Y251 residues, located at the domain interface, playing a central role in interdomain communication. In addition, patch-clamp experiments on GLIC I240A and L241A mutants, located in the M2 helix, demonstrated their importance in channel gating. Together, these results shed light on the sequential and asymmetric conformational transitions that occur during GLIC activation, offering a deeper mechanistic understanding of asymmetric gating in pLGICs.

## Significance

Pentameric ligand-gated ion channels (pLGICs) are essential for fast neurotransmission and are key drug targets for neurological diseases. Although their overall structures are known, how these channels change shape during activation remains unclear due to the fleeting nature of intermediate states. This study uses cryoelectron microscopy combined with meticulous supervised particle alignment to capture high-resolution structures of asymmetric intermediates in the bacterial homopentameric channel GLIC. Molecular dynamics simulations and electrophysiology link these structures to specific functional roles, and targeted mutations identify key residues involved in gating. These findings reveal that even symmetric ion channels can activate through asymmetric pathways, offering insight into pLGIC function and guiding future efforts in drug development.

Pentameric ligand-gated ion channels (pLGICs) have been extensively studied due to their pharmacological significance (1–10). An in-depth understanding of the gating cycle of pLGICs at the molecular level is essential for effectively targeting these channels to develop newer therapeutics. The gating starts with the resting state of pLGICs, which can be captured in an experimental design without an agonist (11–14), while the open/desensitized states can be stabilized after agonist exposure (15–23). The difficulty in capturing intermediate states has limited our understanding of the transitional mechanisms underlying channel gating. The current insights into activation are largely derived from structural comparisons between the resting and open states, showing that the extracellular domain (ECD) undergoes counterclockwise rotation and contraction, involving the rearrangement of several loops in the ECD. These motions subsequently propagate to the transmembrane domain (TMD), leading to the expansion of the channel pore formed by the M2 helices (1, 11, 18, 23–29). Although major functional states such as the resting and open states are well characterized, further studies are needed to investigate the intermediate preactive and desensitized states, which could serve as targets to modulate the equilibrium of activation kinetics. The current approaches to study intermediate states include chemical crosslinking or mutations (30, 31), fluorescent sensors (32, 33), and partial agonists (34–39), which might have undesirable effect on the conformations. Intriguingly, pLGIC activation has been hypothesized to be asymmetric, where conformational transitions of the subunits are asynchronous (21, 40–43). However, detailed structural descriptions of asymmetric activation remain elusive due to the difficulty of resolving subtle asymmetries during reconstruction.

**Fig. 1.**
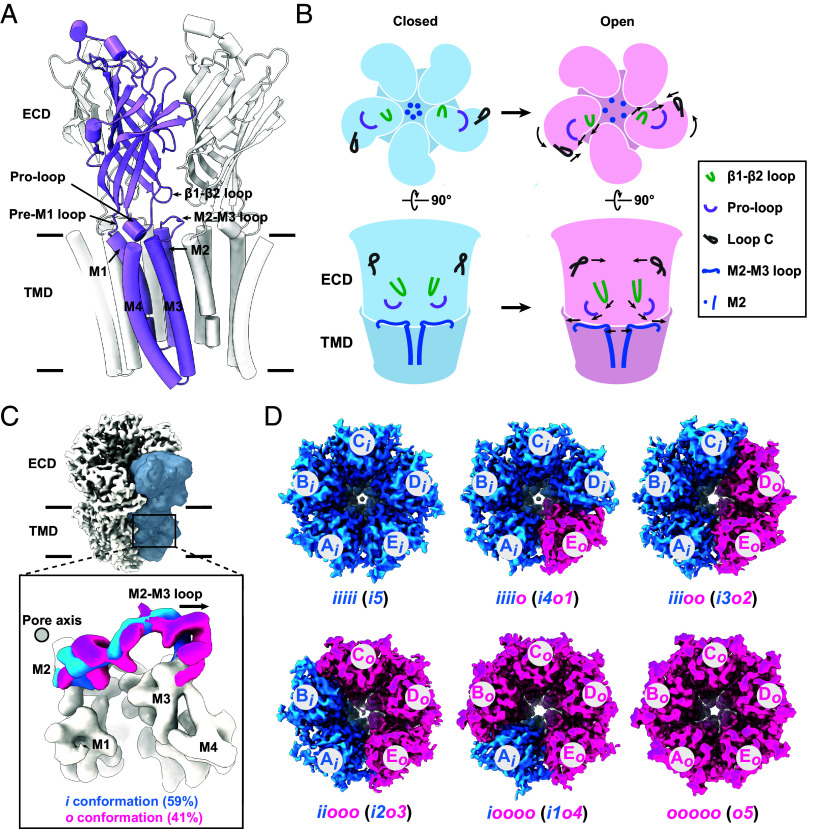
Asymmetric activation of GLIC. (*A*) Overall architecture of GLIC in cartoon representation. One subunit is highlighted in plum for clarity; two additional subunits are shown in gray, while the remaining two are omitted for simplicity. Key structural regions are labeled. (*B*) Schematic illustration of the conformational transition from the closed to open state upon activation. Arrows indicate the direction of movements. A color key for relevant regions is provided in the inset. (*C*) Superposition of cryo-EM density maps corresponding to two distinct M2–M3 loop conformations; inward (*i*; dodger blue) and outward (*o*; deep pink), obtained via focused classification on a single subunit. The mask used for classification (dark gray) is overlaid on the density (light gray). The pore axis is indicated by a solid circle. Population percentages are shown in parentheses. (*D*) High-resolution maps of GLIC in symmetric and asymmetric states are shown in *Top* view, highlighting arrangement of *i* (dodger blue) and *o* (deep pink) conformations in GLIC pH 4.0 dataset. Subunits in the pentamer are labeled clockwise from A to E, with their corresponding M2–M3 loop conformations indicated (e.g., in “*iiiio,*” subunits A–D adopt the i conformation, while subunit E adopts the *o* conformation).

*Gloeobacter violaceus* proton-gated ion channel (GLIC), a prokaryotic model of pLGICs, is particularly well-suited to stabilize preactive states under suboptimal activating conditions modulated by pH (43–45). The GLIC homomer consists of five subunits, each with an ECD and TMD (Fig. 1*A*). GLIC is activated by the protonation of a series of titratable residues in the ECD (40, 46, 47), leading to counterclockwise rotation and contraction of the ECD. This is accompanied by rearrangements of characteristic loops, such as the closure of loop C, the downward displacement and outward movement of the β1–β2 loop and the outward translation of the Pro-loop from the central axis (Fig. 1*B*). These conformational changes propagate to the TMD, resulting in the outward movements of the M2–M3 loop along with the pore-forming M2 helices (44) (Fig. 1*b*). GLIC has yielded only symmetric atomic structures so far at all tested pH conditions (11, 15, 16, 44, 45, 48, 49). However, previous studies reported that GLIC can adopt asymmetric conformations during activation (40, 43), leading to our hypothesis that under subactivation conditions above pH_50_ (pH above 2.9) (50, 51), variations in protonation, may induce subunit-specific conformational differences within a single channel, potentially leading to asymmetric structures. Assuming asymmetric conformations exist in the previous cryo-EM dataset of homomeric GLIC at the subactivation condition (44), the inability to reconstruct them is attributed to the constraints inherent in the alignment technique or classification algorithm in current cryo-EM analysis. The most widely used approach so far involves aligning asymmetric particles and refining them without imposing symmetry, often combined with methods such as symmetry relaxation (27, 52) and focused classifications (28, 29, 53–57). This strategy relies on distinct asymmetric features and has been successfully applied mainly to heteromeric complexes (27–29, 55) or homomeric assemblies with pronounced subunit conformations (21, 53, 54). However, neither condition applies to the homomeric GLIC dataset as differences between subunit conformations are subtle. Therefore, we established a data processing strategy involving symmetry expansion, backtracking particles, supervised particle reorientation, and 3D reconstruction (*Methods*) to resolve high-resolution maps of various asymmetric intermediate states of nanodisc-reconstituted GLIC at pH 4.0, which provide comprehensive understanding of activation mechanism of GLIC. We further used molecular dynamics (MD) simulations to obtain a detailed illustration of their conductive properties. Additionally, our cryo-EM studies revealed the molecular basis for functional roles of Y251 and F116 located at the domain interface and I240 and L241 located at the M2 helix. The role of I240 and L241 in channel opening was further validated using whole-cell patch clamp recordings. Taken together, our cryo-EM results, MD simulations, and functional studies provide a detailed depiction of the asymmetric activation mechanism of GLIC.

## Results

### Identification and 3D Reconstruction of Symmetric and Asymmetric Conformations of GLIC.

In our previous study, three symmetric structures of GLIC at pH 4.0 were resolved, corresponding to the nonconductive preopen C1 and C2 states and a conductive open O state (44). The prominent structural difference among these states was observed at the M2–M3 loop. The position of this loop represents the functional states of GLIC (15, 24, 25, 58, 59). Its inward position toward the central axis represents state toward preopen conformations, while the outward position of this loop from the central axis represents states toward channel opening. The asymmetric conformations were not observed in the cryo-EM reconstruction of GLIC at subactivation pH 4.0 without symmetry imposition, likely due to the averaging of subunit conformations. To gain deeper insights into the structural basis of asymmetric activation in GLIC, we reprocessed our recently reported dataset of nanodisc-reconstituted GLIC at pH 4.0 (44) by combining particles corresponding to the C1, C2, and O states, applying symmetry expansion, and performing classification focused on a single subunit (*SI Appendix*, Fig. S1). As expected, focused classification revealed the most significant structural differences at the TMD. In particular, the M2–M3 loop exhibited either inward (*i*) or outward (*o*) conformations (Fig. 1*C* and *SI Appendix*, Fig. S1). We observed pentamers with multiple distinct subunit conformational arrangements which we refer to as configurations (*Methods*). Further using our data processing approach (*Methods*), six high-resolution asymmetric maps with configurations of *iiiio* (or *i4o1*), *iiioo* (or *i3o2*)*, iiooo* (or *i2o3*)*, ioooo* (or *i1o4*)*, iioio*, and *ioioo* were reconstructed without imposing symmetry with resolutions ranging from 3.08 to 3.45 Å (Fig. 1*D* and *SI Appendix*, Figs. S1 and S2 and Table S1). Similarly, two symmetric maps with configurations of *iiiii* (or *i5*) and *ooooo* (or *o5*) were reconstructed using C5 symmetry with resolution of 2.96 and 2.85 Å, respectively (Fig. 1*D* and *SI Appendix*, Figs. S1 and S2 and Table S1). The *i5* and *o5* configurations were also reconstructed by applying C1 symmetry, which showed marginally reduced resolution compared to imposed C5 symmetry (*SI Appendix*, Fig. S3 and Table S2). All maps display clear densities for both domains, enabling model building for the entire protein (*SI Appendix*, Figs. S2 and S4–S10). The fitted models were validated by generating map-to-model Fourier Shell Correlation (FSC) and by calculating the Q-score for the M2–M3 loop (*SI Appendix*, Figs. S2 and S4–S10 and Table S3). Structural comparison showed that GLIC in *i5* and *o5* conformations resembles the previously resolved preopen C1 and open O states (44), with RMSD_Cα_ of 0.64 Å and 0.18 Å, respectively (*SI Appendix*, Fig. S11 *A* and *B*). Additionally, the positions of annular lipids observed in *i5* and *o5* states are consistent with those observed in C1 and O states (*SI Appendix*, Fig. S11*C*). Notably, lipid 7, equivalent to lipid 9 from our previous study (44), is observed exclusively in the *o* subunit. This supports its proposed role in channel gating, as described in our earlier work (44).

### Conformational Changes at Subunit and Domain Interfaces During Asymmetric Activation.

**Fig. 2.**
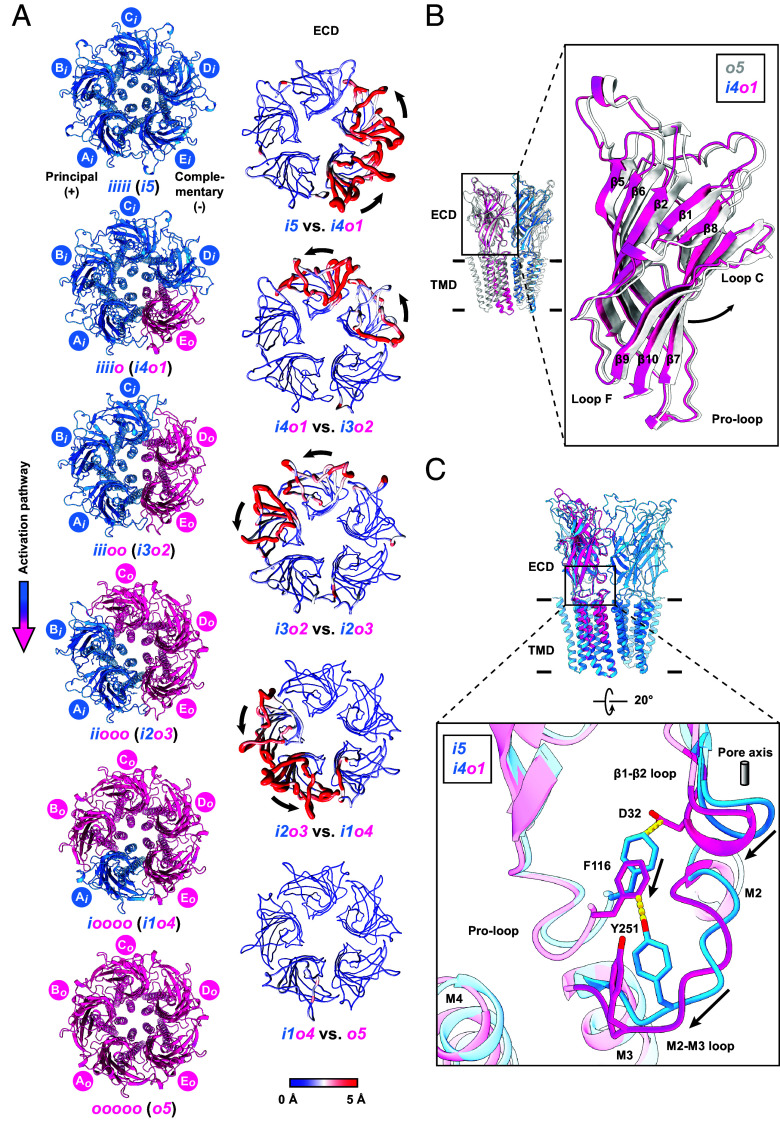
Conformational transitions upon activation in the ECD and at the domain interface. (*A*) Symmetric and asymmetric atomic models of GLIC in various configurations are shown in *Top* view as cartoons. RMSD_Cα_ between configurations are represented as a putty plot (*Top* view), highlighting conformational changes in the ECD. The TMD is omitted for clarity. Arrows indicate the direction of movement. A color key corresponds to RMSD_Cα_. (*B*) Superposition of the overall GLIC structures in the *o5* (gray) and *i4o1* (deep pink) states. *Inset* shows a zoomed-in view of the ECD of subunit E. (*C*) Superposition of GLIC in the *i5* (dodger blue) and *i4o1* (deep pink) states. The zoomed-in view highlights a downward displacement of the β1–β2 loop in *o* conformation, which causes steric clashes between D32 (in *i4o1*) and F116 (in *i5*), and between F116 (in *i4o1*) and Y251 (in *i5*). For clarity only one clash symbol is shown (yellow dashed). D32, F116, and Y251 are shown as sticks and colored by heteroatom. Arrows indicate directions of displacement. The pore axis is represented as a gray cylinder.

To understand the activation mechanism of GLIC, we compared *i5* with *i4o1*, showing a counterclockwise rotation of the ECD in subunit E(*o*) (Fig. 2*A*). The upper part of the ECD in subunit E(*o*) of the *i4o1* state shows incomplete rotation compared to any subunit in the *o5* state, indicating an intermediate conformation (Fig. 2*B*). The RMSD_Cα_ calculated between *i5* and other states (*i3o2, i2o3, i1o4,* and *o5*) indicated that the activation of one subunit (*o* conformation) triggers conformational changes in the ECD of its complementary subunit. These changes then propagate in a sequential manner until all subunits transition to the *o* conformation (Fig. 2*A*). To understand the mechanism of signal transduction from ECD to TMD, we superimposed the *i5* and *i4o1* states. In the *i4o1* state, the β1–β2 loop in the E(*o*) subunit shows a downward displacement and lateral translation from the central axis. This movement allows a salt bridge to form between D32 in β1–β2 loop and R192 on the Pre-M1 loop (*SI Appendix*, Fig. S12*A*), a key interaction previously shown to stabilize the open state (46, 47). The rearrangement of the β1–β2 loop introduces steric clashes between D32 (Fig. 2*C*, deep pink) and the bulky aromatic side chain of F116 (Fig. 2*C*, dodger blue), located in the Pro-loop (post-β6 loop). These clashes displace the Pro-loop away from the central axis (Fig. 2*C*). The positional shift of F116 (Fig. 2*C*, deep pink) induces an outward translation of Y251, located at the M2–M3 loop (Fig. 2*C*, deep pink). This is evidenced by a potential steric clash if Y251’s conformation were to remain unaltered (Fig. 2*C*, dodger blue). The coordinated outward displacement of the Pro-loop and M2–M3 loop maintains hydrogen bonding interactions between the backbone of F116 and the side chain of Y251 (*SI Appendix*, Fig. S12*B*). The downward displacement of the β1–β2 loop and the concomitant rearrangement of Pro-loop in the *o* conformation are consistently associated with outward displacement of the M2–M3 loop during subunit activation. Thus, our structures show that steric clashes and hydrogen bonding interactions at the domain interface play a critical role in channel activation.

### Gradual Pore Widening and Ion Permeation Mechanism.

**Fig. 3.**
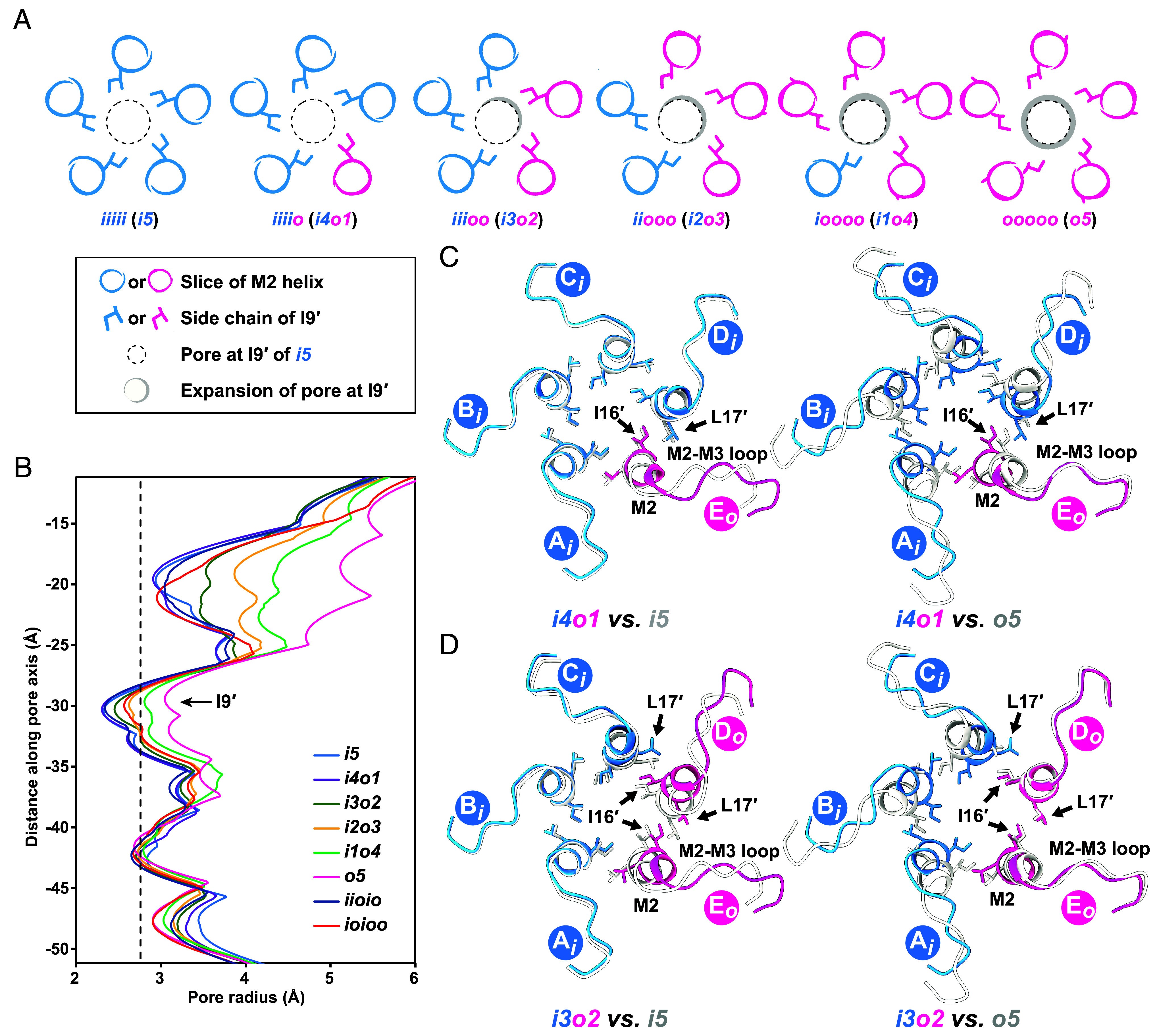
Conformational rearrangement in TMD. (*A*) Sliced top views of the pore-lining M2 helices in various asymmetric states, illustrating sequential pore expansion. A key is provided in the *Inset*. The I9′ is shown as sticks. Subunits adopting the *i* and *o* conformations are colored dodger blue and deep pink, respectively. (*B*) Pore radius profiles of TMD plotted against the distance along the pore axis, comparing different states. The black dashed line indicates the approximate radius of a hydrated Na^+^ ion. The position of I9′ is indicated by arrow. (*C*) Sliced *Top* views showing superpositions of the M2 helix and M2–M3 loop in *i4o1* with *i5* (gray, *Left*) and with *o5* (gray, *Right*). (*D*) Sliced top views showing superpositions of the M2 helix and M2–M3 loop in *i3o2* with *i5* (gray, *Left*) and with *o5* (gray, *Right*). I16′ and I17′ are shown as sticks. Subunits in *i* and *o* conformations are colored as in panel a (dodger blue and deep pink, respectively).

In all our structures, the TMD, particularly the M2 helix and the M2–M3 loop, displays the highest-quality density maps, with local resolution estimated to be better than 2.5 Å (*SI Appendix*, Figs. S2 and S13*A*). To understand the mechanism of channel opening, the ion permeation pathway of symmetric and asymmetric GLIC structures were compared. The pore radius of the *o5* state is wider than that of *i5* which agrees with the previous comparison between the O and C1 states of GLIC (*SI Appendix*, Fig. S12*C*). Further comparison of asymmetric states revealed a gradual pore widening upon activation, most prominently at the hydrophobic gate I9′ (Fig. 3 *A* and *B*). While the M2–M3 loop in the E(*o*) subunit of the *i4o1* state exhibited an outward displacement compared to *i5*, the constriction at I9′ remained largely unchanged (Fig. 3 *A* and *B*). Although the top part of the M2 helix displaces away from the central axis, the lower portion particularly around I9′ exhibits minimal conformational change. Detailed analysis revealed that L241 (L17′) in the M2 helix of the complementary D(*i*) subunit sterically hinders the outward movement of I240 (I16′) in the principal E(*o*) subunit, thereby restricting its conformational change (Fig. 3*C*). Subsequently, when subunit D adopts *o* conformation (*i3o2*), I16′ of subunit E(*o*) and L17′ of subunit D(*o*) undergo radial displacements compared to *i4o1* state. This is likely driven by the combined influence of subunit D activation and conformational drive exerted by principal subunit E(*o*) (Fig. 3*D*). Similarly, the radial displacement of I16′ in subunit D(*o*) drives L17′ of subunit C away from the pore axis (Fig. 3*D*). This effect propagates sequentially across the subunits, eventually leading to the adoption of the *o* conformation along with maximum expansion of I16′ and L17′ in all subunits (*SI Appendix*, Fig. S13 *B* and *C*). The changes in pore radius of *i4o1* compared to *i5* state is indistinguishable, whereas the pore gradually expands from *i3o2* state onward and maximum expansion occurs in the *o5* state (Fig. 3 *A* and *B*).

**Fig. 4.**
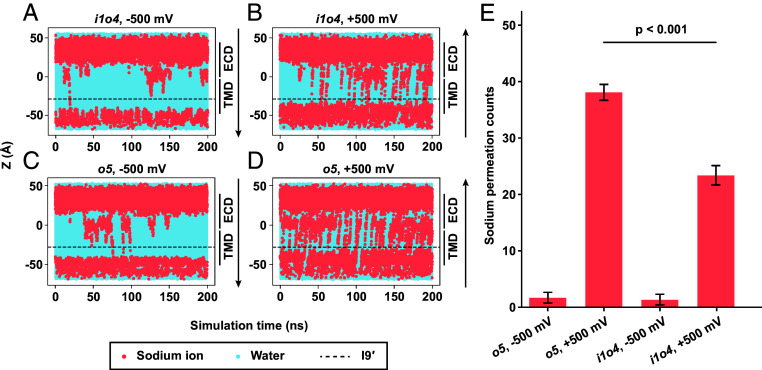
Ion permeation MD simulations performed in the presence of transmembrane potentials. Results for *i1o4* with an external electric field of −500 mV (*A*) or +500 mV (*B*), compared with results of *o5* under −500 mV (*C*) and +500 mV (*D*), reveal that *i1o4* is partially conductive. The sodium ions and water molecules are colored in salmon and blue, respectively. The positions of ECD and TMD are indicated on the right of each plot. The position of I9′ is indicated by a dashed line. The vertical arrows show the directions of ion passage. (*E*) The bar plot of ion permeation events for *i1o4* and *o5*, with ±500 mV potential differences. The error bar indicates SD of permeation counts from three repeats; the *P*-value for *o5* and *i1o4* at +500 mV is reported from Welch’s *t* test.

The pore profile of the *o5* state closely resembles that of our previously resolved open state (*SI Appendix*, Fig. S12*C*), suggesting that the *o5* state represents an open, conductive conformation. In the *i4o1*, *i3o2,* and *i2o3* states, the pore radii at I9′ are narrower than the hydrated Na^+^ ion (2.76 Å) (60), indicating nonconductive states (Fig. 3*B*). Interestingly, *i1o4* has a marginally wider pore than the radius of the hydrated Na^+^ ion (Fig. 3*B*). To assign the functional state of *i1o4* in comparison to *o5*, molecular dynamics simulations of the two states were performed. The atomic models were embedded in lipid bilayers containing 50% DOPE, 25% POPC, and 25% POPS, mimicking the cryo-EM sample environment. The simulations predicted stable pore radii without any major changes during 200 ns equilibration (*SI Appendix*, Fig. S14 *A* and *C*). The pore at I9′ is observed to be wetted during the entire simulation, supported by the absence of the energetic barrier for water molecules at that region (*SI Appendix*, Fig. S14 *B* and *D*). MD simulations under external potentials show a similar wetted environment around I9′ during the entire simulation time (Fig. 4 *A*–*D*). Both *i1o4* (Fig. 4 *A* and *B* and *SI Appendix*, Fig. S15) and *o5* (Fig. 4 *C* and *D* and *SI Appendix*, Fig. S16) showed sodium permeation events in both field directions. Notably, a significantly higher number of ion permeation events was observed under a positive transmembrane potential (Fig. 4*E*). This phenomenon has also been reported previously (61), but precise conductance measurements can be challenging because they depend on the extent of protonation of the proton sensors (62). However, at least under these conditions, *i1o4* is statistically significantly less conductive than *o5* (Fig. 4*E*), suggesting that it is a partially conductive state.

### Mutations at Domain Interface Reveal a Crucial Signal Transduction Pathway.

**Fig. 5.**
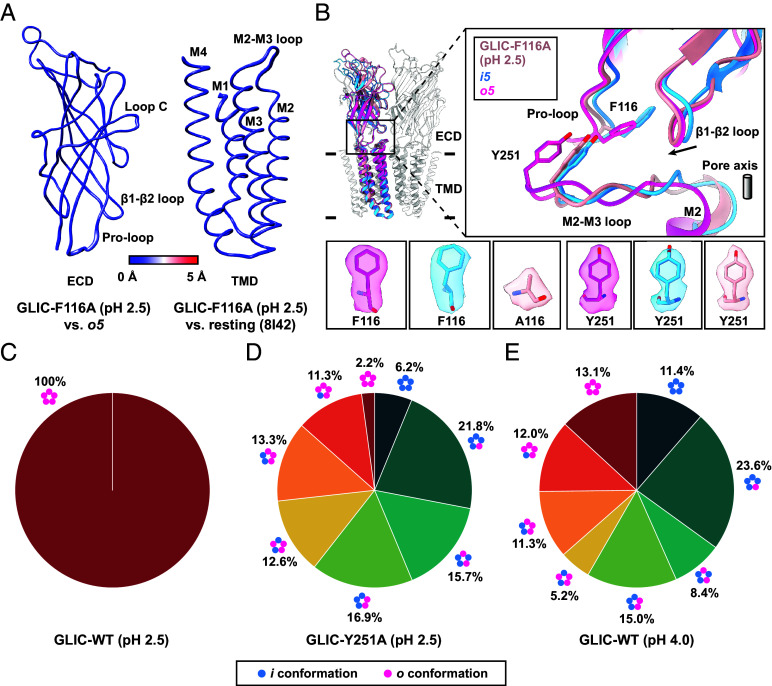
Role of F116A and Y251A mutants in signal transduction. (*A*) Pairwise structural comparisons of the F116A mutant, the ECD is compared with the *o5* state (*Left*), and the TMD is compared with the resting state (PDB: 8I42, *Right*), shown as RMSD_Cα_-based putty representations. (*B*) Superposition of GLIC-F116A mutant (rosy brown), *i5* (dodger blue), and *o5* (deep pink) states shown as cartoons. A zoomed-in view highlights the conformation of F116/A116 in Pro-loop and Y251 in M2–M3 loop. Key residues—F116, Y251, and A116 (in the F116A mutant)—are shown as sticks with corresponding cryo-EM densities. Distributions of configurations of the GLIC-WT at pH 2.5 (*C*), GLIC-Y251A mutant at pH 2.5 (*D*), and GLIC-WT at pH 4.0 (*E*) are shown as pie charts.

Previous functional studies showed that both F116 and Y251 are indispensable for ion conduction (63, 64). Therefore, we generated F116A and Y251A mutants for cryo-EM study. The GLIC-F116A was reconstituted into nanodiscs at pH 2.5, a condition that stabilizes wild-type (WT) channels in an open conformation (44). The 3D classifications focused on a single subunit confirmed that all subunits exhibit *i* conformation. Therefore, the maps were reconstructed using both C1 and C5 symmetry with a nominal resolution of 2.53 and 2.14 Å, respectively (*SI Appendix*, Figs. S3 and S17). The reconstructed maps show clear densities at various regions (*SI Appendix*, Fig. S18). The atomic model built into the cryo-EM map was validated by generating map-to-model Fourier Shell Correlation (FSC) and by calculating the Q-score for the M2–M3 loop (*SI Appendix*, Fig. S17 and Table S3). The structure shows an ECD conformation resembling the *o5* state with RMSD_Cα_ of 0.28 Å, while the TMD closely aligns with the previously resolved resting state with RMSD_Cα_ of 0.39 Å (Fig. 5*A*). In the GLIC-F116A mutant, although the β1–β2 loop shows downward displacement (Fig. 5*B*), due to the absence of bulky side chain of F116 supported by a clear alanine density, the Pro-loop shows only subtle outward displacement, leaving the conformation of M2–M3 loop unaffected (Fig. 5*B*). This indicates that the bulky side chain of F116 is required for the outward displacement of the M2–M3 loop. Therefore, this structure highlights the critical role of F116 in transmitting ECD rearrangements to the TMD.

To further understand whether Y251 is the primary residue in the M2–M3 loop responsible for its displacement influenced by F116, we first analyzed the existing crystal structure of GLIC-Y251A mutant (GLIC-Y251A_crystal_, PDB: 4LMK) (64) solved at pH 4.0. The crystal structure represents a nonconducting state with an open-like ECD (PDB: 4HFI, RMSD_Cα_ of 0.56 Å) (48) and a TMD resembling the resting state (PDB: 4NPQ, RMSD_Cα_ of 0.40 Å) (11) (*SI Appendix*, Fig. S19*A*). Despite overall similarities in the ECD, the β1–β2 loop does not exhibit downward displacement during ECD activation (*SI Appendix*, Fig. S19*B*), warranting further investigation. To explore this, we prepared a nanodisc-embedded GLIC-Y251A mutant at pH 2.5 for cryo-EM analysis. Although only a homopentameric configuration was previously resolved at this pH for the WT (Fig. 5*C*) (44), focused classification of GLIC-Y251A mutant unexpectedly revealed that 44% of the subunits adopt the *o* conformation, while the remaining subunits adopt the *i* conformation (*SI Appendix*, Fig. S20). Using a similar data processing strategy, all eight possible configurations were successfully identified (Fig. 5*D* and *SI Appendix*, Fig. S20). Interestingly, only 2.2% of the population adopt the *o5* state whereas 11.3% of the population adopt *i1o4* state (Fig. 5*D* and *SI Appendix*, Fig. S20). Particles corresponding to *i1o4* state were reconstructed to a map with nominal resolution of 2.87 Å, with clear densities across all regions (*SI Appendix*, Figs. S20 and S21). Comparison of the atomic model of GLIC-Y251A *i1o4* built into the cryo-EM map with GLIC-WT *i1o4* shows similar conformation with backbone RMSD_Cα_ of 0.40 Å and similar pore profile (*SI Appendix*, Fig. S22 *A* and *B*). Therefore, the functional profile of GLIC-Y251A *i1o4* should resemble the GLIC-WT *i1o4* which is partially conductive. Similarly, the minor population of GLIC-Y251A *o5* is expected to adopt an open conductive conformation. The pronounced reduction of the *o5* state in Y251A, even at pH 2.5, compared to WT at pH 4.0 (Fig. 5 *D* and *E*), indicates a drastic shift in activation equilibrium. This suggests that Y251 is essential for stabilizing the outward conformation of the M2–M3 loop in coordination with the outward displacement of F116.

### I16′ and L17′ Are Important for Gating Manifested by Electrophysiology.

**Fig. 6.**
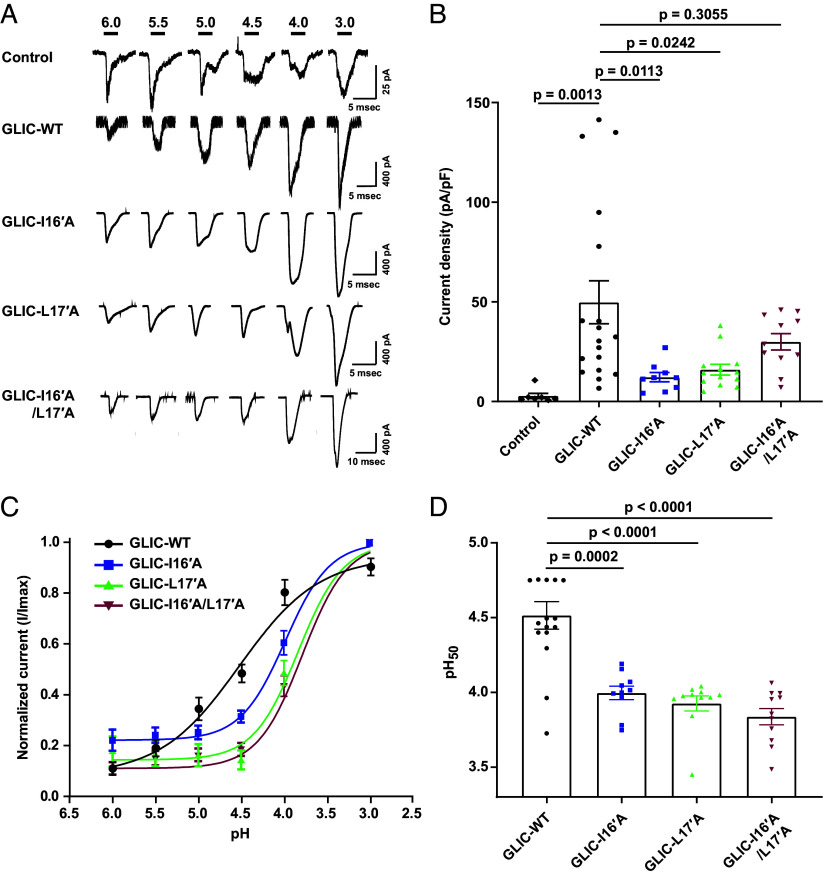
Functional activation pattern of GLIC currents in HEK293 cells. (*A*) Representative whole cell currents at indicated pH values obtained either from control HEK-293 T cells or transfected with GLIC-WT, and its mutants I16′A, L17′A, and I16′A/L17′A cDNAs. Cells were held at −70 mV and the indicated pH solution was applied for 1 to 2 s using a fast perfusion system. (*B*) Bar plots depict mean ± SEM of current density values obtained from control HEK-293 (n = 7), GLIC-WT (n = 18), I16′A (n = 9), L17′A (n = 13), and I16′A/L17′A (n = 11) at pH 4.0. (*C*) The pH_50_ curves of GLIC-WT (n = 16) and mutants I16′A (n = 10), L17′A (n = 11), I16′A/L17′A (n = 11) plotted by sigmoidal dose–response fit to the normalized currents (I/Imax) obtained at various pH solutions. (*D*) The bar plot represents mean ± SEM of pH_50_ values of GLIC-WT (n = 16) and its mutants I16′A (n = 10), L17′A (n = 11), I16′A/L17′A (n = 11). The statistical significance was calculated using one-way ANOVA followed by post hoc Dunnett’s test and pairwise *P* values obtained are represented above respective plots.

Our cryo-EM data revealed that I16′ and L17′ residues in the M2 helix play an important role in pore opening. To validate this observation, we performed mutagenesis (I16′A and L17′A) and conducted whole-cell patch-clamp recordings in HEK293 cells. The gating profile for the WT and mutants show predictable fast activation followed by desensitization indicated by slow decay at activating pH conditions (Fig. 6*A*). Analysis of current density revealed that both single mutants displayed reduced current compared to WT channels (Fig. 6*B*). To test this further, we generated an I16′A/L17′A double mutant, which also produced reduced current upon activation relative to WT (Fig. 6 *A* and *B*). The dose–response curve shows a rightward shift in the mutants compared to the WT (Fig. 6*C*). The pH_50_ of WT was measured to be 4.51 (Fig. 6 *C* and *D*). The reduced currents were associated with decreased pH_50_ values of 3.99, 3.92, and 3.84 for the I16′A, L17′A, and I16′A/L17′A mutants, respectively (Fig. 6 *B* and *D*). Therefore, these functional studies demonstrate the important role of I16′ and L17′ in gating.

## Discussion

Several recent studies have demonstrated that homomeric ion channels can adopt asymmetric conformations (21, 40, 43, 54, 65–68). The focused classification on subunit followed by the identification of corresponding particles has thus far been an exercise in identifying asymmetric conformations with subunit-level asymmetry (21, 56, 57, 69, 70). However, further 3D reconstruction to generate asymmetric cryo-EM maps directly from these identified particles remained challenging especially when the subunit asymmetry results from subtle structural differences. In this study, we analyzed homopentameric GLIC under subactivation conditions to examine structural asymmetry at the molecular level. The eight configurations represent the possible states for the cyclic pentameric assemblies containing two distinct subunit conformations (*SI Appendix*, Fig. S1). Although additional asymmetries may exist in GLIC, particularly within the ECD, we have focused on reporting the discrete states that could be reliably distinguished based on well-resolved conformational differences in the M2–M3 loop. Our data processing scheme, especially the particle reorientation within the same configuration, significantly assisted the precise 3D reconstruction of asymmetric particles present in cryo-EM data which yielded several high-resolution asymmetric structures of GLIC reaffirming previous reports (40, 43). Moreover, asymmetric GLIC structures at various activation stages offer detailed molecular insights into the gating mechanism (43). Notably, among all the structures resolved at pH 4.0, the unexpected presence of the *i5* state suggests that activation begins with a symmetric rotation of the ECD from resting state, which is followed by the onset of asymmetric rotation. This finding is consistent with the previous observation where a fast transition to the preopen intermediate state followed by slower and localized transition for channel opening has been proposed (32). Our asymmetric structures also provided an opportunity for detailed investigation of the molecular basis of the propagation of conformation changes from ECD to TMD. Alignments across subunits revealed that F116, located in the Pro-loop, plays a central role in communicating the structural changes between the β1–β2 loop in the ECD and the M2–M3 loop in the TMD. This observation led us to propose a steric-driven mechanism underlying the conformational transitions. The mutations of F116 and Y251 have been reported to be nonfunctional, implying their critical role in channel gating (63). Our results show that the ECD of the GLIC-F116A mutant adopts an open-like conformation, while the TMD remains in a resting-like state, indicating the channel is arrested in a preopen intermediate conformation. A previous crystal structure of Y251A resolved only a state with activated ECD and closed pore (63, 64). Our cryo-EM data yielded unexpected results with 86.5% nonconductive channels, 11.3% partially conductive channels and only 2.2% conductive channels, which suggests that the Y251A mutation reduces the channel conductivity significantly. These findings suggest that Y251 plays a crucial role in gating by stabilizing the outward M2–M3 loop conformation (*o*).

Analysis based on a previous phylogenetic study (71) showed that Y251 is conserved in 58% of pLGICs, whereas F116 shows conservation in 15% of pLGICs (*SI Appendix*, Fig. S23*A*). The Y251 equivalent residue is observed in the α1 glycine receptor (Y303) and α1/β2/ρ1 GABA_A_ receptors (Y282/Y277/Y340) (*SI Appendix*, Fig. S23*B*). The steric clashes between the equivalent phenylalanine and tyrosine residues are similarly conserved in these receptors, as observed in GLIC (*SI Appendix*, Fig. S23*B*). The Y303C mutation in the *GLRA1* gene is a well-identified cause of startle syndrome or hyperekplexia, where the EC_50_ for glycine spikes 70-folds and maximal currents decrease 50-folds (72, 73). Similarly, Y277C mutation in *GABRB2* or *GABRB3* lead to epilepsy (74–76). The association of these mutations with neurological disorders underscores the crucial role of these residues. Here, our study elucidates the structural basis by which these residues modulate the activation equilibrium through the reconstruction of high-resolution asymmetric preopen states for GLIC. Although phenylalanine is not well conserved in most neurotransmitter receptors, a leucine residue takes its place in both α1 glycine receptor and GABA_A_ receptors, which may also sterically couple to the tyrosine on the M2–M3 loop upon the downward displacement of the β1–β2 loop (*SI Appendix*, Fig. S23*B*); however, this needs further investigations.

The multiple asymmetric states also enabled us to investigate the key role of I16′ and L17′ in the M2 helix in channel gating. In the *i4o1* state, the outward displacement of the M2–M3 loop in subunit E(*o*) does not affect the pore radius (Fig. 3*A*). This suggests that activation of subunit E(*o*) alone does not generate sufficient force to induce conformational changes in the M2 helix of subunit D(*i*). However, this scenario changes when subunit D is activated and adopts the *o* conformation (*i3o2*). In this state, L17′ in the M2 helix of subunit D(*o*) is already under steric strain from I16′ of subunit E(*o*). This strain is further amplified by the activation of subunit D. The combined effect of these forces causes radial displacement of L17′, resulting in outward displacement of the M2 helix in subunit D(*o*) along with I16′ in subunit E(*o*) as indicated by the expanded pore (Fig. 3 *A* and *B*). This process continues as additional complementary subunits transition to the *o* conformation, ultimately leading to complete pore opening. This highlights a cooperative mechanism in channel activation that is validated by whole-cell patch-clamp recordings of mutants showing a pH_50_ shift toward more acidic conditions compared to WT. While these two residues play a significant role, mutating these residues does not abolish channel function, suggesting that additional contributing forces are involved in facilitating pore opening.

In addition to multiple asymmetric states, we also resolved diagonally activated *iioio* and *ioioo* states. Similar states have previously been observed in the homopentameric 5-HT_3A_ receptor (21). However, the roles of the *iioio* and *ioioo* conformations within the activation pathway require further investigation. On a cautionary note, the eight resolved states were captured on a cryo-EM grid rather than from a time-resolved experiment. Therefore, the proposed sequence of channel activation should be considered as a working model, and the definitive sequence of events remains to be determined. The major insight from this study is that the closure of a single subunit can have a significant impact on the overall channel function of GLIC. This is a crucial observation, as it suggests that targeting a single subunit, for example, through a drug or antagonist may be sufficient to reduce the channel activity significantly. Notably, prior research has shown that propofol can bind asymmetrically to the TMD of GLIC, inducing asymmetric structural changes that result in channel closure (77). These findings highlight the importance of the interplay between symmetric and asymmetric conformations in the rapid modulation of ion channel conductance. This concept aligns with previous observations in various enzyme systems, where subtle deviations from perfect symmetry were found to be essential for dynamic functional activity (78). Building on the previous 5-HT_3A_R study (21), which suggested that symmetry loss could be a general principle of ion channel activation, our work provides a platform for further investigation of such asymmetry in other enzymes and ion channels. In this regard, our data processing approach provides a powerful framework for probing the functional role of structural asymmetry, as it can be broadly applied to macromolecules with cyclic pseudosymmetry.

## Methods

### Protein Expression and Purification of GLIC-WT.

GLIC was expressed and purified as reported previously (44). Briefly, GLIC-MBP was recombinantly expressed in *Escherichia coli* C41 cells grown in TB with 50 μg/mL kanamycin at 37 °C until OD_600_ reached 0.8, followed by induction with 200 μM IPTG at 18 °C for 14 to 16 h. Cells were lysed in ice-cold buffer (20 mM HEPES pH 7.5, 100 mM NaCl, 1 mM Phenylmethylsulfonyl fluoride (PMSF), 1 µM Leupeptin, and 1 µM Pepstatin), and membranes were isolated by ultracentrifugation (168,000×*g*, 45 min). The membranes were solubilized in 1% n-dodecyl-β-D-maltoside (DDM), and cleared lysate after ultracentrifugation was incubated with amylose resin (2 mL) at 4 °C for 1.5 to 2 h. After washing, GLIC-MBP was eluted with buffer containing 20 mM HEPES pH 7.5, 100 mM NaCl, 20 mM Maltose, and 0.05% DDM and concentrated to 8 to 10 mg/mL. The MBP tag was cleaved with HRV-3C protease, and GLIC was purified by size exclusion chromatography using the Superdex 200 Increase 10/300 column equilibrated with buffer containing 20 mM HEPES pH 7.5, 100 mM NaCl, 0.025% DDM.

### Site-Directed Mutagenesis, Protein Expression, and Purification of F116A & Y251A Mutants.

To generate the F116A mutant, the primers 5′-CGCTGGATGCTCGCCGCTATCCTTTT-3′ (forward) and 5′-GGCGAGCATCCAGCGGCGAGAG-3′ (reverse) were used. For the Y251A mutant, the primers 5′-ACCCCCGCCATGACCTACACGG-3′ (forward) and 5′-GGTCATGGCGGGGGTCTTTGGAAG-3′ (reverse) were used. The point mutations were confirmed by sequencing and the plasmids were transformed into *E. coli* C41 cells for protein expression. Cells were grown in TB media for 16-18 h after induction with 200 μM IPTG and harvested by 4,000×*g* centrifugation and lysed using Avestin Emulsiflex C3 homogenizer. Membrane fraction was separated by centrifugation at 168,000×*g* for 45 min at 4 °C and the pellet was resuspended into buffer A containing 20 mM HEPES pH 7.5, 100 mM NaCl.

For protein purification, the resuspended membrane was solubilized using DDM, followed by ultracentrifugation at 168,000×*g* for 15 min at 4 °C to remove insoluble debris. The supernatant was incubated with 2 mL of amylose resin (NEB) at 4 °C for 1.5 to 2 h. The mixture was then transferred to a gravity flow column. The resin was washed with 40 mL of buffer B (buffer A+1 mM EDTA and 0.05% DDM), and the bound protein was eluted with 10 mL of elution buffer (buffer B plus 20 mM maltose). The eluted protein was concentrated to a final concentration of 8 to 10 mg/mL using Amicon Ultra-4 centrifugal filter units with a 100 kDa molecular weight cutoff. The MBP tag was subsequently cleaved using HRV-3C protease. The resulting mixture containing digested GLIC, MBP, and protease was separated by size exclusion chromatography (SEC) on a Superdex 200 Increase 10/300 column (GE Healthcare), pre-equilibrated with buffer containing 20 mM HEPES pH 7.5, 150 mM NaCl, 0.025% DDM, using a Cytiva ÄKTA purifier system.

### Reconstitution of GLIC in Lipid-Nanodisc.

1,2-dioleoyl-sn-glycero-3-phosphoethanolamine (DOPE), 1-palmitoyl-2-oleoyl-glycero-3-phosphocholine (POPC), and 1-palmitoyl-2-oleoyl-sn-glycero-3-phospho-L-serine (POPS) (from Avanti Research) were used for nanodisc preparation due to their stability at low pH for cryo-EM studies as mentioned previously (79). These lipids were mixed in a 2:1:1 molar ratio and dried under a stream of nitrogen gas to form a thin film followed by resuspension in buffer A. Purified protein (F116A or Y251A mutants), lipids, and MSP1E3D1 were mixed in the molar ratio of 1:3:360. The mixture was gently rotated overnight at 4 °C in the presence of approximately 10 mg/0.5 mL of Bio-Beads SM-2 adsorbents (Bio-Rad Laboratories, Cat. No. 1523920) to remove detergents. The nanodisc-reconstituted protein was purified using Superdex 200 Increase 10/300 column, pre-equilibrated with buffer containing 10 mM sodium citrate pH 4.0 and 150 mM NaCl.

### Cryo-EM Sample Preparation and Data Collection.

Purified nanodisc-reconstituted proteins were concentrated to approximately 0.5 mg/mL, followed by three rounds of buffer exchange with a buffer containing 10 mM sodium citrate buffer pH 2.5 and 150 mM NaCl using Amicon Ultra-4 centrifugal filters with a 100 kDa molecular weight cutoff. Grids were prepared by applying 3.5 μL sample onto freshly plasma cleaned graphene-coated grids (GLIC-F116A: Au 300, R1.2/1.3 Quantifoil; GLIC-Y251A: Cu 300, R1.2/1.3 Quantifoil) at 100% humidity and temperature of 4 °C followed by plunge-freezing in liquid ethane using the FEI Vitrobot Mark IV (Thermo Fisher Scientific).

The data were collected using FEI Titan Krios (Thermo Fisher) transmission electron microscope operating at 300 kV, equipped with a Falcon 4i direct electron detector and a Selectris X energy filter using a 10 eV slit width. For the F116A and Y251A mutants, 11,131 and 10,188 movies were collected, respectively, at a nominal magnification of 165,000× (pixel size of 0.76 Å). Data were collected using a total electron dose of 70e^−^/Å² and a defocus range of 0.5 to 1.5 μm (*SI Appendix*, Table S1).

### Cryo-EM Data Processing of GLIC-WT at pH 4.0.

For each individual dataset, raw movies were motion-corrected by MotionCor 2 (v1.6.3) (80). Contrast Transfer Function (CTF) was estimated by Ctffind4 (v4.1.13) (81) or using patch CTF in cryoSPARC (v4.3.1) (82). We reanalyzed our previous cryo-EM dataset of GLIC-WT at pH 4.0 (44). A total of 511,578 particles obtained from this dataset were used for 3D refinement in RELION (v4.0.1) (83). Subsequently, symmetry expansion was performed using *relion_particle_symmetry_expand*, generating 2,557,890 particles. We created a single subunit mask using the *molmap* function in ChimeraX (84) which was used to generate single subunit initial map. This initial map was low-pass filtered to 15 Å for focused 3D classifications and refinements. Using T-value of 16 in 3D classification, we identified inward (*i*) and outward (*o*) M2–M3 loop conformations. A total of 1,838,537 particles corresponding to *i* and *o* conformations were selected for further analysis, while particles with unresolved loop conformations were excluded. To determine the distribution of *i* and *o* conformations within pentamers, the 3D classification metadata file (run_data.star) was analyzed to extract each particle’s coordinates (_rlnCoordinateX, _rlnCoordinateY), rotational angle of subunits within the pentamer (_rlnAngleRot), and subunit conformation (_rlnClassNumber). Using this metadata, we calculated statistics on arrangement of *i* and *o* conformations within pentamers using *configurations_stats.sh* script. The results generated from script revealed all eight possible configurations of *i* and *o* conformations within the pentamer: *iiiii (i5), iiiio (i4o1), iiioo (i3o2), iiooo (i2o3), ioooo (i1o4), ooooo (o5), iioio,* and *ioioo*, with population frequencies ranging from 5.2 to 23.6% (*SI Appendix*, Fig. S1).

To generate symmetric 3D reconstructions, particles from symmetric classes (*i5* and *o5*) were sorted and concatenated into single (*.star*) files which were further used in 3D refinement with C5 symmetry, resulting in density maps with nominal resolutions of 2.96 Å and 2.85 Å, respectively. These maps were used for atomic model building. Both maps were also reconstructed without imposing symmetry for comparison. For asymmetric reconstructions, particles were grouped by configuration, which represent pentamer with combination of *i/o* subunits. Since, particles in the same configuration may have multiple orientations (euler angles), a precise alignment was required to avoid averaging of the asymmetric features during refinement. To address this, we developed, *align.sh*, a script designed for particles with cyclic pseudosymmetry to select a specified configuration and reorient them into a consistent orientation based on focused classification results. The aligned particle sets were refined in RELION using either a local angular search (±15°) or by reconstructing without additional alignment, both of which produced 3D maps with no change in resolution. The maps for asymmetric configuration showed a resolution range of 3.08 to 3.45 Å which were used for atomic model building (*SI Appendix*, Fig. S1). The final set of particles was used to generate angular distribution plots in cryoSPARC.

### Cryo-EM Data Processing of GLIC F116A and Y251A Mutants.

For F116A data, 2,039,213 particles were picked using blob and template based particle picking. Iterative 2D classifications, ab-initio, and heterogeneous refinement were performed in cryoSPARC. Further, 1,267,247 particles were exported to RELION and subjected to another round of 3D classification without alignment and refinement (*SI Appendix*, Fig. S17). To identify conformation of M2–M3 loop at subunit level, all the particles were subjected to symmetry expansion and focused classification as described earlier. The focused classifications demonstrated that all classes adopt inward conformation of the M2–M3 loop, indicating that asymmetric reconstruction was no longer necessary. Therefore, 1,267,247 particles were subjected to iterative 3D classifications, 3D refinements using C5 symmetry, Bayesian polishing and postprocessing, resulting in the final map with nominal resolution of 2.14 Å (*SI Appendix*, Fig. S17). The final cryo-EM map was also reconstructed without imposing symmetry for statistical comparison with the asymmetric structures. Similarly, for the Y251A dataset, 1,782,582 particles were picked using blob and template based particle picking. Iterative 2D classifications, ab-initio, and heterogeneous refinement were performed in cryoSPARC (*SI Appendix*, Fig. S20). Further, 923,377 particles were exported to RELION and subjected to symmetry expansion and focused classification, which showed subunits with inward (55.9%) and outward (44.1%) M2–M3 loop conformations (*SI Appendix*, Fig. S20). These subunits were found to be arranged in eight possible configurations using *conformations_stats.sh* script (*SI Appendix*, Fig. S20). Only 6.2 and 2.2% of particles belong to the *i5* and *o5* state, respectively. The remaining 91.6% particles adopt asymmetric configurations. Among all the configurations, the particles belonging to *i1o4* state were further used for 3D reconstruction, resulting in a map with a nominal resolution of 2.87 Å (*SI Appendix*, Fig. S20).

### Model Building and Refinement.

The cryo-EM structures of GLIC in the closed (PDB ID: 8I48) or open (PDB ID: 8WCR) states were used as initial models for model building (*SI Appendix*, Table S1). These models were first aligned to our density maps using UCSF ChimeraX and subsequently built manually in Coot (v0.9.8.7) (85). Real-space refinement was performed using the phenix.real_space_refine tool from the PHENIX package (v1.21rc1-4985) (86), with default settings, except for enabling full NCS constraints, refining NCS operators fully, and setting the “max reasonable bond distance” to 500. The stereochemistry of the final models was assessed using the MolProbity web server (v4.5.2) (87). The Q-scores are calculated by the Mapq plugin of Chimera (88) using each final postprocessed map and the corresponding model as inputs. Pore profiles were calculated using the HOLE program (v2.2.005) (89). All structural figures were generated using ChimeraX (v1.7) and finalized in Adobe Illustrator.

### Molecular Dynamics Simulations.

Cryo-EM structures of GLIC *i1o4* and *o5* states were embedded in bilayer membranes composed of DOPE, POPS, and POPC at a 2:1:1 ratio, using the CHARMM-GUI Membrane Builder (90–94), within simulation cells of 11.6 × 11.6 × 15.8 nm^3^, which were then solvated and neutralized with 150 mM NaCl. Protein and lipids were described with the CHARMM36m force field (95). The chosen water model was TIP3P (96), and water molecules were constrained with the SETTLE algorithm (97). GLIC being a proton-gated channel, protonation state assignment is of particular importance. In a number of previous studies, several acidic residues (E26, E35, E67, E75, E82, D86, D88, E177, and E243) were protonated, and H277 was doubly protonated to replicate low pH conditions, but this protonation scheme is not independently confirmed (41). Our observations indicate that E67, E82, D86, and D88 interfere with Na^+^ ion entry into the ECD, and based on PROPKA3 predictions (98), we left these residues deprotonated in our simulations. Molecular dynamics (MD) simulations were performed with GROMACS 2021.5 (79, 99–105), using a 2 fs integration time-step. Bonds to hydrogen atoms were constrained via the LINCS algorithm. Long-range electrostatics were handled with the smooth Particle Mesh Ewald method (106). Simulations were maintained at 310 K and 1 bar using a velocity-rescaling thermostat (107) and a semi-isotropic rescaling barostat (108), with coupling constants of 1 and 5 ps, respectively. To preserve the conformational integrity of each cryo-EM structure, an extended equilibration protocol was used to prepare all structures for simulation. First, harmonic position restraints on protein heavy atoms were gradually released over several equilibration steps. Then, flat-bottom harmonic cross-distance restraints were applied between selected Cα atoms of pore-lining residues (Thr20′, Ile16′, Ala13′, Ile9′, Ser6′, Thr2′, Glu−2′) to maintain pore conformation and level of openness in an approach based on work by Dämgen and Biggin (109). These restraints activated only if Cα atoms moved closer than in their original EM structures, with a force of 5,000 kJ mol^−^^1^ nm^−^^1^. This restrained equilibration was run for 200 ns. Water free-energy profiles were calculated for various conformations using the Channel Annotation Package (110). Simulation trajectories were analyzed at 100 ps intervals with a 0.14 nm bandwidth for water density estimation. For the *i1o4* and *o5* structures, the ion conduction was assessed by applying a +500 mV and −500 mV transmembrane potential in 200 ns simulations, using a uniform electric field along the membrane normal.

### cDNAs and Molecular Cloning.

GLIC-WT and its mutants (I240A, L241A, I240A/L241A) complementary DNAs (cDNAs) in pTLNXA7 were subcloned into pcDNA6 vector for overexpression in HEK293 cells. The open reading frame (ORF) of GLIC-WT and mutants were PCR amplified and cloned to pcDNA6 vector digested with KpnI and AgeI restriction enzymes with the help of the ClonExpress II One Step Cloning kit (Vazyme), following the manufacturer’s instructions. The forward and reverse primers used for subcloning of wild-type and mutant cDNAs are 5′-CGTTTAAACTTAAGCTTGGTACCGCCACCATGGGACTGAGAG-3′ and 5′-GTGATGGTGATGATGACCGGTTTAAAATCCAAAGAAAAGAAATGCCAG-3′, respectively. *E. coli* top10 cells were used for the cloning and amplification of all the cDNAs.

### Cell Culture and Transfection.

Adherent HEK293 cells were grown on high glucose containing Dulbecco’s Modified Eagle Medium (DMEM) supplemented with 10% fetal bovine serum and 1% penicillin/streptomycin and maintained at 37 °C with 5% CO2 in a humidifier environment as performed previously (111, 112). For the electrophysiological experiments cells were seeded with 50 to 60% confluency on 12 mm round glass coverslips placed in 35 mm dishes. After 12 h of plating, cells were transiently transfected with 1.5 µg of either GLIC-WT or mutant cDNAs in pcDNA6 backbone along with cotransfection of eGFP for identification of transfected cells.

### Whole-Cell Patch-Clamp Recordings.

The whole-cell patch-clamp recordings were obtained from transiently transfected HEK293 (tsA201) cells using a MultiClamp 700B computer-controlled amplifier operated in conjunction with Digidata 1550B digitizer for data digitization run on pClamp 11 software package (Molecular Devices, USA). Cells were bathed with buffer solution consisting of 140 mM NaCl, 2.8 mM KCl, 2 mM CaCl_2_, 2 mM MgCl_2_, 10 mM HEPES, and 10 mM Glucose adjusted to pH 7.4 with NaOH. For attaining pH 7.0, a similar HEPES buffer was used. For pH 6.0, pH 5.5, pH 5.0, and pH 4.5 equimolar MOPS was used, and the pH was adjusted by adding NaOH or HCl. For pH 3.0 and 4.0, trisodium citrate and citric acid-based buffers were used, keeping all the salt composition same as mentioned before. Thick-walled glass pipettes (Sutter Instruments, USA) made of borosilicate were used for patching the transfected HEK293 cells either with GLIC-WT or mutant cDNAs to record the macroscopic currents under whole cell configuration at room temperature (22 to 24 °C) after 36 h of transfection. The pipette solution consisted of 140 mM KCl, 5 mM MgCl_2_, 5 mM EGTA, 10 mM HEPES, 5 mM Phosphocreatine, 2 mM Mg-ATP, 0.5 mM Na-GTP adjusted to pH 7.3 with NaOH. After attaining whole-cell mode, cells were held at –70 mV and currents were recorded for GLIC-WT and mutant by quick solution exchange from pH 7.4 to different pH solutions using a computer controlled 8-channel gravity perfusion system (Warner Instruments, USA). The pH solution was exposed for 1 to 2 s to estimate the peak for activation kinetics. To estimate desensitization kinetics pH solution was exposed for 10 to 20 s until the desensitization curve was saturated or reached baseline. In between pH shifts the cells were washed with a pH 7.4 control solution for recovery.

### Electrophysiology Analysis.

The electrophysiological recordings obtained from pClamp software were analyzed with the Clampfit module (Molecular Devices, USA). The current density of GLIC-WT and mutant channels were obtained by normalizing the peak current amplitudes with the capacitance of respective cell recordings of pH 4.0. The average of the normalized current (I/Imax) and SEM calculated using GraphPad Prism 8.0, were used to plot dose–response curves in Origin 8.5. Dose–response sigmoidal fit function was used to calculate pH_50_, as follows:

$$y=A1+[(A2-A1)/(1+10^{\left(\text{LOG}x0-x\right)p})].$$

Bar plot was generated using pH_50_ values calculated from dose–response curves. The statistical significance was determined using one-way ANOVA, followed by post hoc Dunnett’s T3 test for multiple comparisons between GLIC-WT and its mutants. The pairwise *P* values obtained were represented in the plots.

## Supplementary Material

Appendix 01 (PDF)

## Data Availability

Accession Nos.: The coordinates are deposited with PDB ID 9LAG (*iiiii*) (113), 9LAI (*iiiio*) (114), 9LAJ (*iiioo*) (115), 9LAK (*iiooo*) (116), 9LB9 (*ioooo*) (117), 9LBA (*ooooo*) (118), 9LBB (*iioio*) (119), 9LBC (*ioioo*) (120), 9LBD (GLIC-F116A) (121), 9LBE (*ioooo_Y251A_*) (122), respectively. Corresponding maps are deposited with EMDB IDs: 62927 (*iiiii*) (123), 62929 (*iiiio*) (124), 62930 (*iiioo*) (125), 62931 (*iiooo*) (126), 62937 (*ioooo*) (127), 62938 (*ooooo*) (128), 62939 (*iioio*) (129), 62940 (*ioioo*) (130), 62941 (GLIC-F116A) (131), 62942 (*ioooo_Y251A_*) (132), respectively. All simulation boxes, input files and final frames are available on Zenodo (https://doi.org/10.5281/zenodo.15322266) (133). All the scripts are deposited in GitHub (https://github.com/SandipLab/asymReconstruct.git) (134). All other data are included in the manuscript and/or *SI Appendix*.
