# Supplementary material for: Asymmetric gating of a homopentameric ion channel GLIC revealed by cryo-EM
Source: Proc Natl Acad Sci U S A. 2025 Oct 23;122(43):e2512811122. doi: 10.1073/pnas.2512811122 (PMC12582304; doi:10.1073/pnas.2512811122)
Supplement: Supplementary file 1 — Appendix 01 (PDF) [file pnas.2512811122.sapp.pdf]

PNAS

Supporting Information for
Asymmetric gating of a homopentameric ion channel GLIC revealed by cryo-EM

Zhuowen Li[1], Nikhil Bharambe[1], Kashmiri Manishrao Lande[3]*, Bjarne Feddersen[4]*, Asha Manikkoth Balakrishna[1], Philip C. Biggin[4], Giriraj Sahu[3], and Sandip Basak[1,2#]

[1]School of Biological Sciences, Nanyang Technological University, Singapore 637551
[2]NTU Institute of Structural Biology, Nanyang Technological University, Singapore 639798
[3]Molecular Biophysics Unit (MBU), Indian Institute of Science, India
[4]Structural Bioinformatics and Computational Biochemistry, Department of Biochemistry, University of Oxford, Oxford, UK

*These authors contributed equally to this work.

[#]Corresponding author: Sandip Basak, School of Biological Sciences, Nanyang Technological University, Singapore 637551. Phone: +65 69082204.
Email: sandip.basak@ntu.edu.sg

This PDF file includes:

Figures S1 to S23
Tables S1 to S3

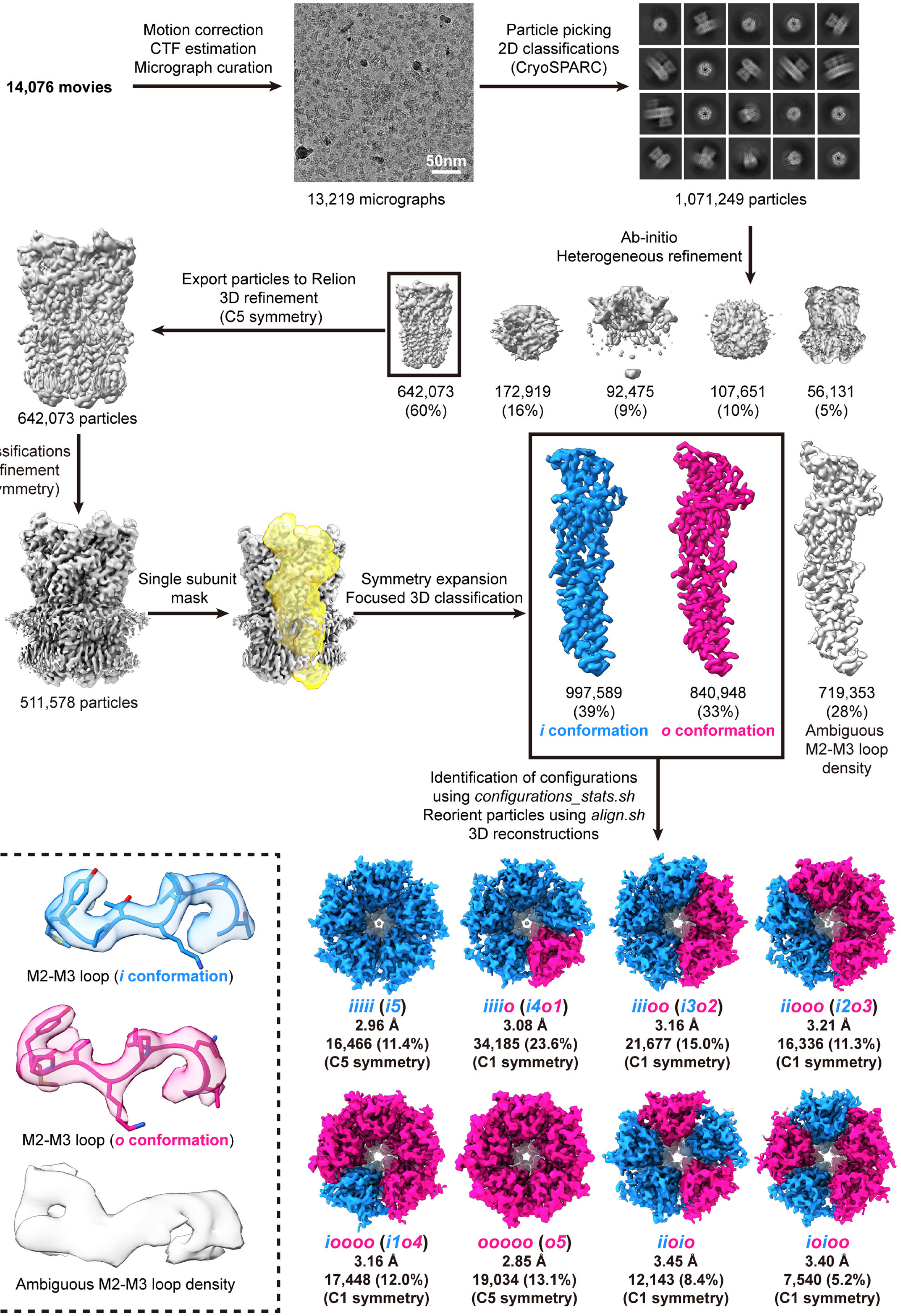

14,076 movies
Motion correction
CTF estimation
Micrograph curation
50nm
13,219 micrographs
Particle picking
2D classifications
(CryoSPARC)
1,071,249 particles
Ab-initio
Heterogeneous refinement
642,073
(60%)
172,919
(16%)
92,475
(9%)
107,651
(10%)
56,131
(5%)
Export particles to Relion
3D refinement
(C5 symmetry)
642,073 particles
3D classifications
3D refinement
(C5 symmetry)
511,578 particles
Single subunit
mask
Symmetry expansion
Focused 3D classification
997,589
(39%)
i conformation
840,948
(33%)
o conformation
719,353
(28%)
Ambiguous
M2-M3 loop
density
Identification of configurations
using configurations_stats.sh
Reorient particles using align.sh
3D reconstructions
M2-M3 loop (i conformation)
M2-M3 loop (o conformation)
Ambiguous M2-M3 loop density
iiiii (i5)
2.96 Å
16,466 (11.4%)
(C5 symmetry)
iiiio (i4o1)
3.08 Å
34,185 (23.6%)
(C1 symmetry)
iiioo (i3o2)
3.16 Å
21,677 (15.0%)
(C1 symmetry)
iiooo (i2o3)
3.21 Å
16,336 (11.3%)
(C1 symmetry)
ioooo (i1o4)
3.16 Å
17,448 (12.0%)
(C1 symmetry)
ooooo (o5)
2.85 Å
19,034 (13.1%)
(C5 symmetry)
iioio
3.45 Å
12,143 (8.4%)
(C1 symmetry)
ioioo
3.40 Å
7,540 (5.2%)
(C1 symmetry)

Supplementary Figure 1. Cryo-EM data re-processing workflow of GLIC at pH 4.0. Cryo-EM data processing workflow for GLIC at pH 4.0, including representative micrograph, 2D class averages, ab-initio reconstruction, heterogeneous refinement, and non-uniform refinement. Particles were subjected to symmetry expansion followed by focused classification using a single subunit mask (gold, transparent). Focused classification revealed M2–M3 loop conformations as either inward (*i*) or outward (*o*), while a subset of particles exhibited ambiguous density in this region, which is shown in the dashed-line box. The following symmetric and asymmetric states were obtained: *iiiii*, *iiiio*, *iiioo*, *iiooo*, *ioooo*, *ooooo*, *iioio*, and *ioioo*, with corresponding nominal resolutions of 2.96 Å, 3.08 Å, 3.16 Å, 3.21 Å, 3.16 Å, 2.85 Å, 3.45 Å, and 3.40 Å, respectively.

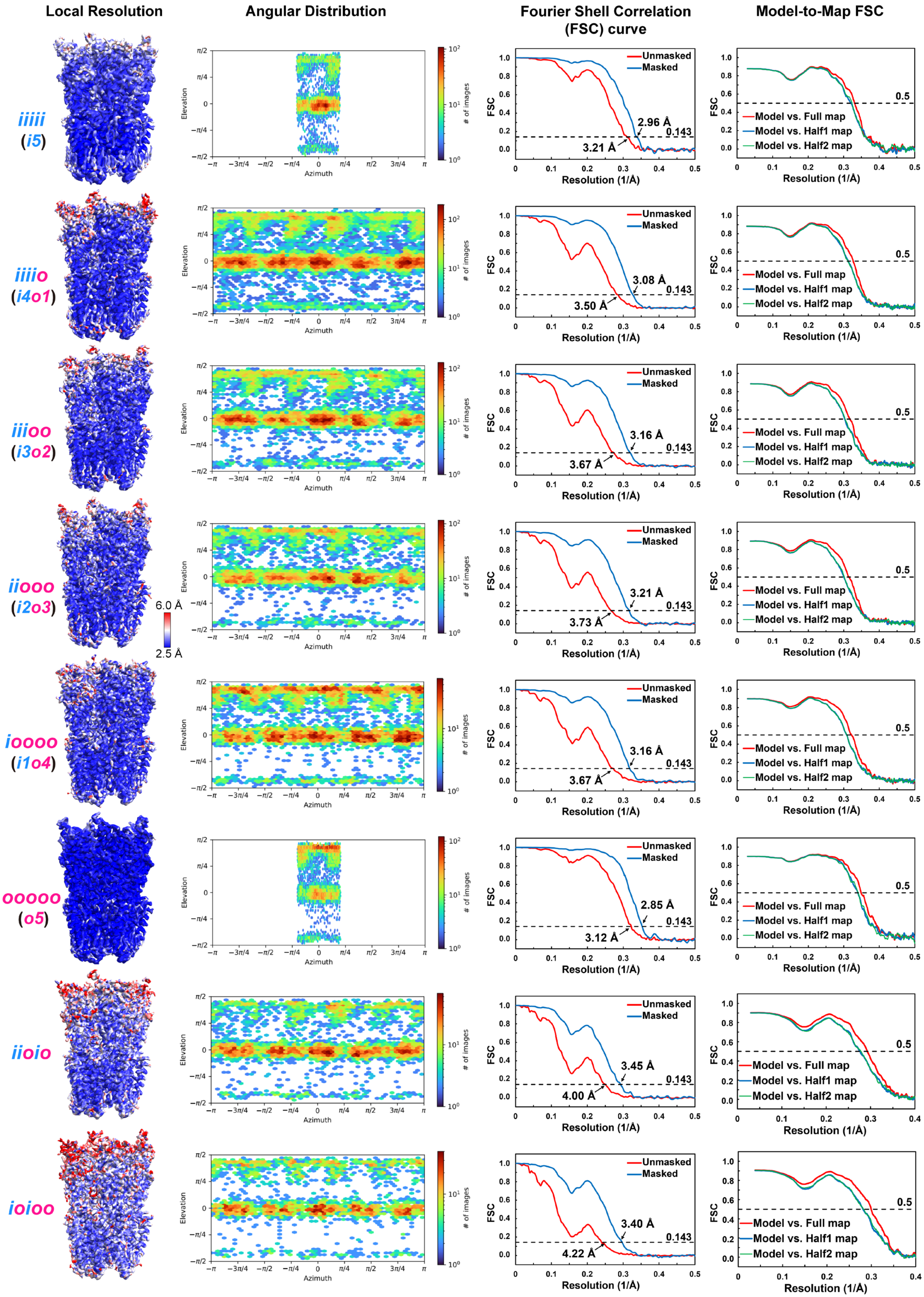
Local Resolution
Angular Distribution
Fourier Shell Correlation (FSC) curve
Model-to-Map FSC
iiiii (i5)
iiiio (i4o1)
iiioo (i3o2)
iiooo (i2o3)
ioooo (i1o4)
ooooo (o5)
iioio
ioioo
6.0 Å
2.5 Å
Elevation
Azimuth
# of images
FSC
Resolution (1/Å)
Unmasked
Masked
0.143
0.5
Model vs. Full map
Model vs. Half1 map
Model vs. Half2 map
2.96 Å
3.21 Å
3.08 Å
3.50 Å
3.16 Å
3.67 Å
3.21 Å
3.73 Å
3.16 Å
3.67 Å
2.85 Å
3.12 Å
3.45 Å
4.00 Å
3.40 Å
4.22 Å

Supplementary Figure 2. Gold-standard FSC, angular distribution, local resolution, and model-to-map validation. Gold-standard Fourier Shell Correlation (FSC), angular distribution, local resolution estimation, and model-to-map validation FSC curves are shown for the *iiiii*, *iiiio*, *iiioo*, *iiooo*, *ioooo*, *ooooo*, *iioio*, and *ioioo* states. The FSC curves before (red) and after (blue) masking are displayed for each map, with the dashed line indicating the 0.143 FSC cutoff. Angular distributions were calculated using cryoSPARC. Side views of the corresponding 3D reconstructions are colored by local resolution as estimated with ResMap. A resolution color key is provided for reference.

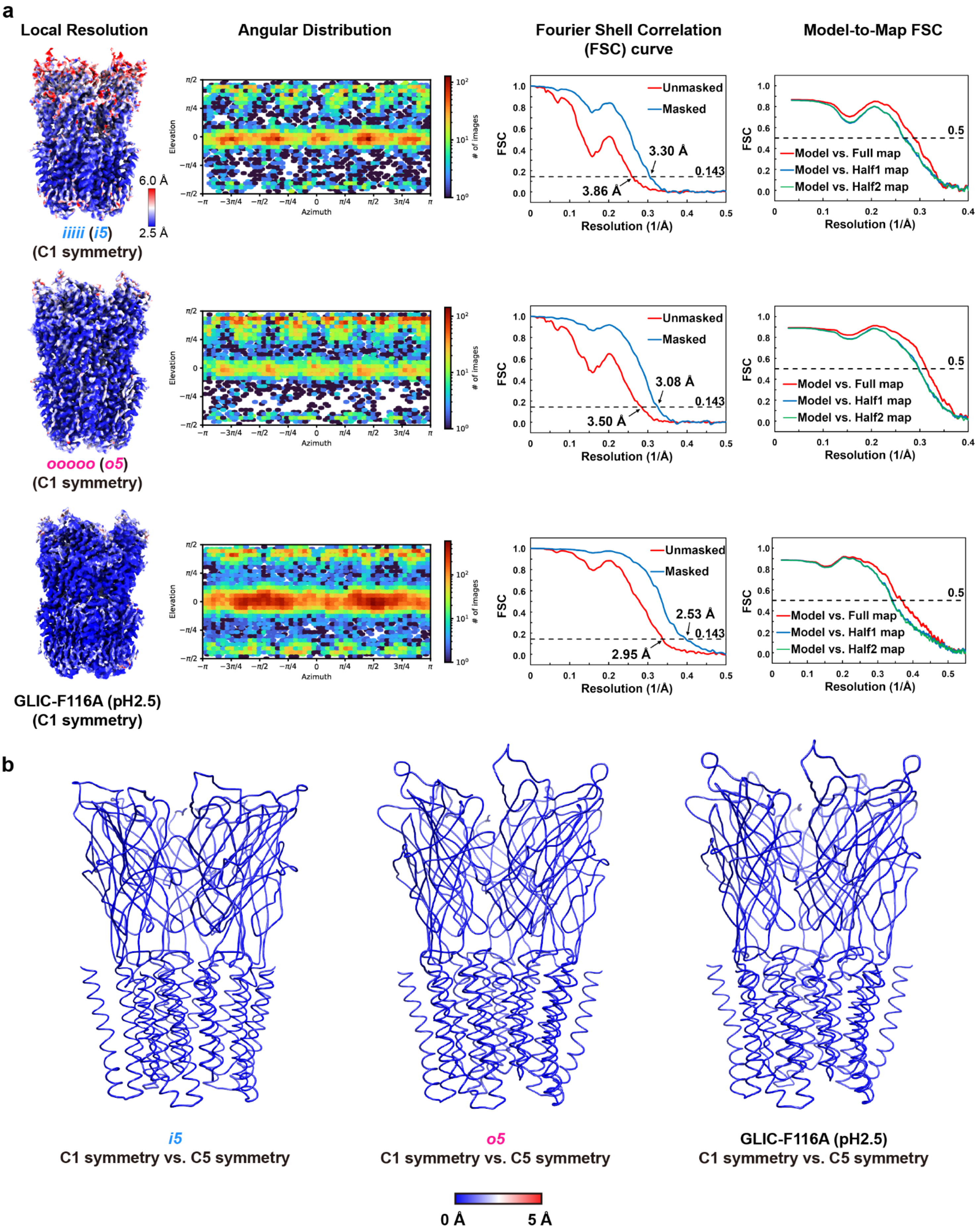
a
Local Resolution
Angular Distribution
Fourier Shell Correlation (FSC) curve
Model-to-Map FSC
6.0 Å
2.5 Å
iiiii (i5)
(C1 symmetry)
Elevation
Azimuth
# of images
FSC
Unmasked
Masked
3.30 Å
0.143
3.86 Å
Resolution (1/Å)
0.5
Model vs. Full map
Model vs. Half1 map
Model vs. Half2 map
ooooo (o5)
(C1 symmetry)
3.08 Å
3.50 Å
GLIC-F116A (pH2.5)
(C1 symmetry)
2.53 Å
2.95 Å
b
i5
C1 symmetry vs. C5 symmetry
o5
C1 symmetry vs. C5 symmetry
GLIC-F116A (pH2.5)
C1 symmetry vs. C5 symmetry
0 Å
5 Å

Supplementary Figure 3. The refinements of *i5, o5,* and GLIC-F116A structures with C1 symmetry. a Gold-standard FSC, angular distribution, local resolution, and model-to-map validations for *i5*, *o5*, and GLIC-F116A structures refined with C1 symmetry. The FSC curves before (red) and after (blue) masking are displayed for each map, with the dashed line indicating the 0.143 FSC cutoff. Angular distributions were calculated using cryoSPARC. Side views of the corresponding 3D reconstructions are colored by local resolution as estimated with ResMap. A resolution color key is provided for reference. **b** Pairwise $RMSD_{C\alpha}$ comparisons between the C5 and C1 symmetry refinements for *i5*, *o5*, and GLIC-F116A at pH 2.5. A distance color key is provided for reference.

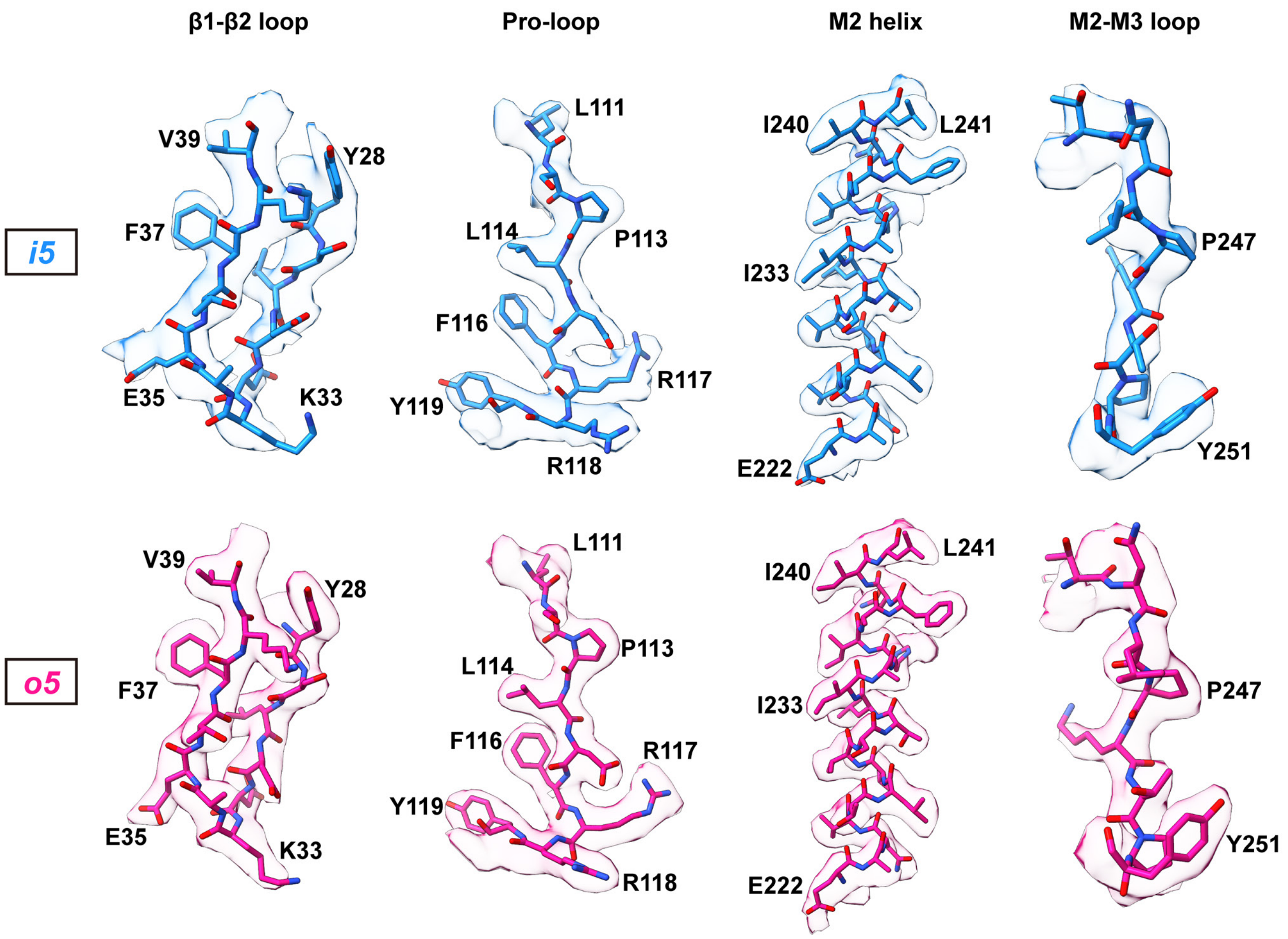

Supplementary Figure 4. Validation of densities in various regions in the *i5* and *o5* states. Density map validation of key structural regions—β1–β2 loop, Pro-loop, M2 helix, and M2–M3 loop—is shown for the *i5* (dodger blue) and *o5* (deep pink) states. The corresponding model regions are shown as stick representations overlaid with semi-transparent density maps, colored to match each conformation.

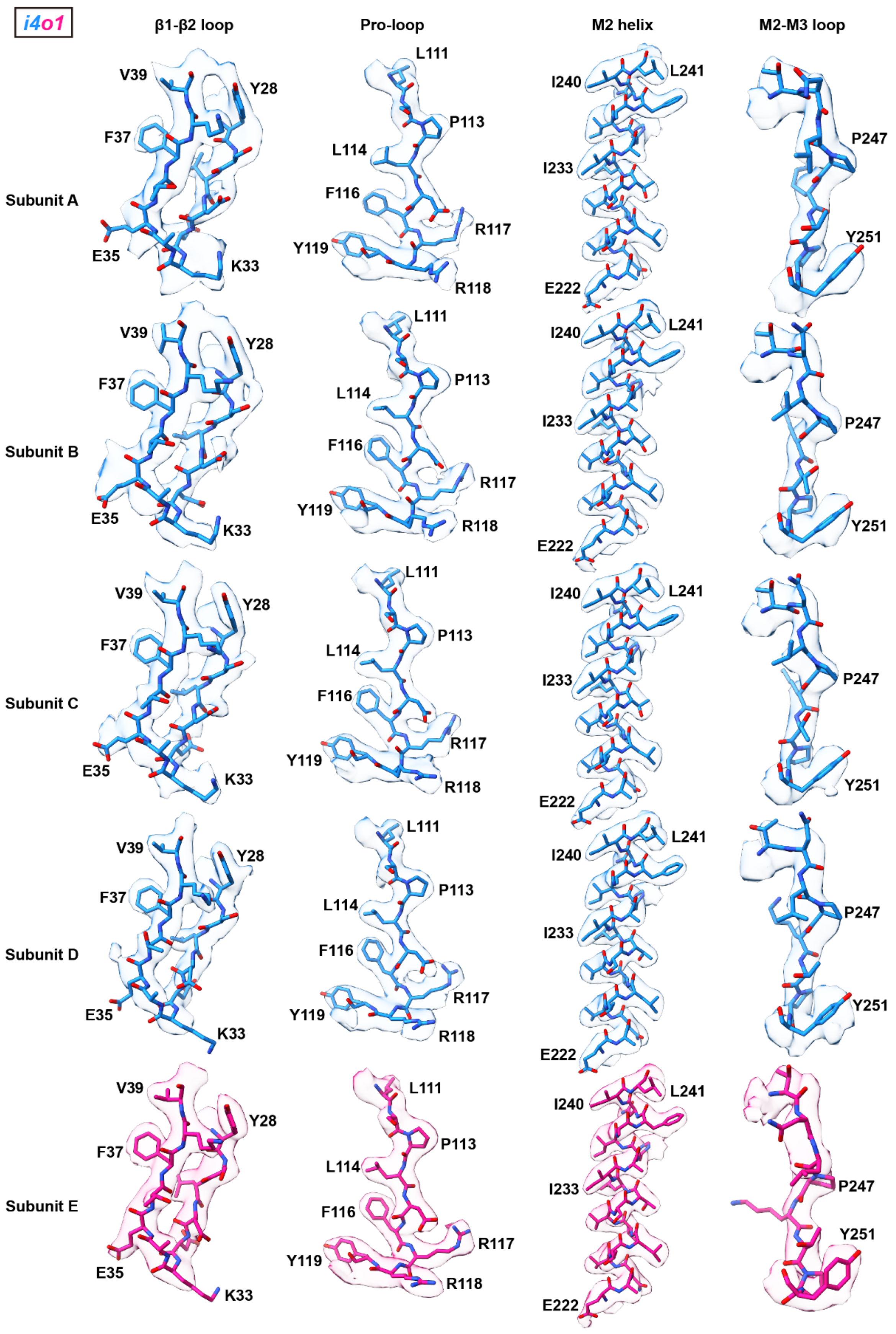
i4o1
β1-β2 loop
Pro-loop
M2 helix
M2-M3 loop
Subunit A
Subunit B
Subunit C
Subunit D
Subunit E
V39
Y28
F37
E35
K33
L111
P113
L114
F116
R117
Y119
R118
I240
L241
I233
E222
P247
Y251

Supplementary Figure 5. Density validation of various regions in the *i4o1* state.

Density maps of key structural regions—β1–β2 loop, Pro-loop, M2 helix, and M2–M3 loop—are shown for subunits A through E in the *i4o1* state. Subunits in the *i* and *o* conformations are colored dodger blue and deep pink, respectively. Corresponding model segments are depicted as sticks overlaid with semi-transparent density maps, colored to match each conformation.

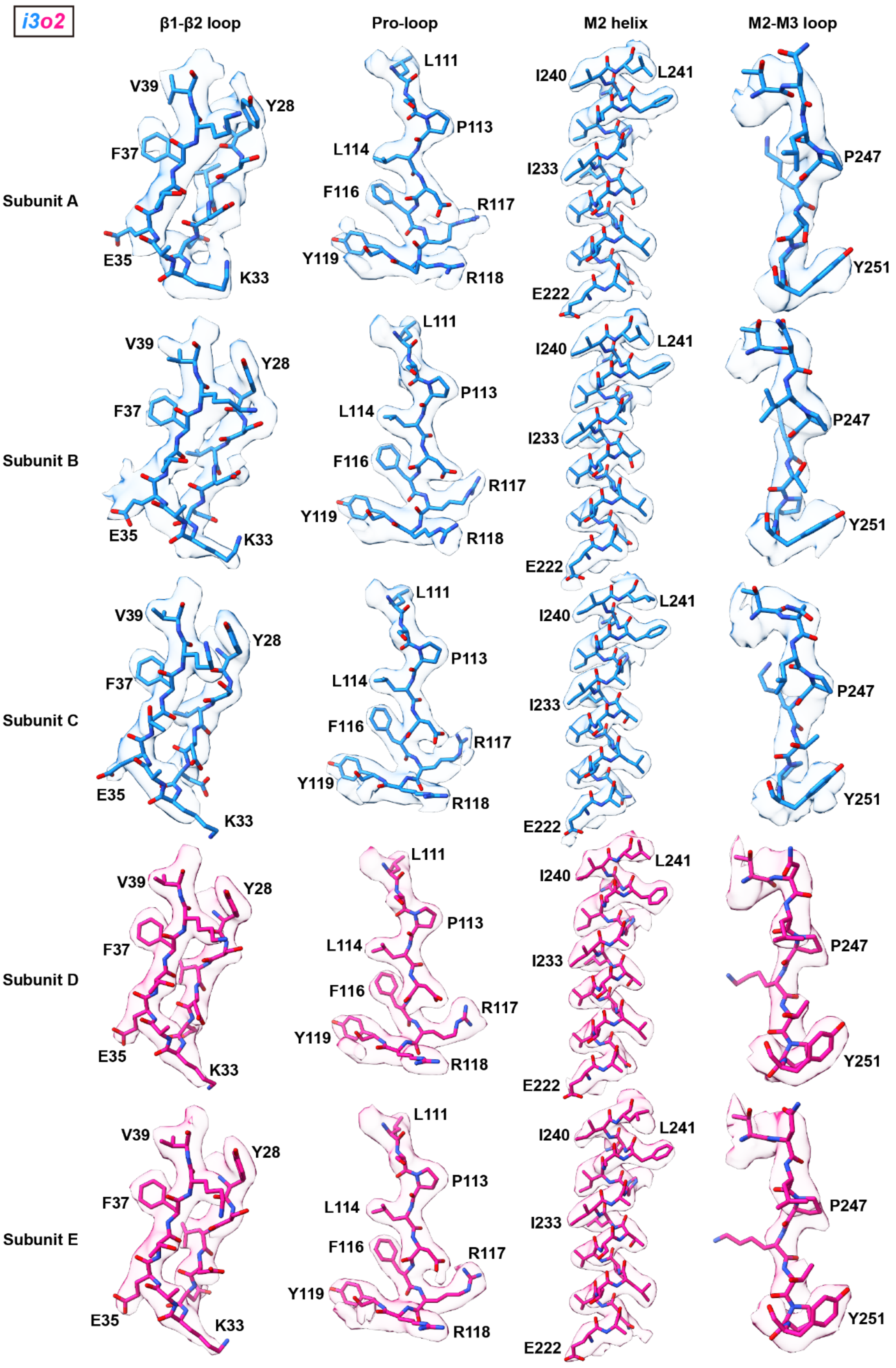

i3o2
β1-β2 loop
Pro-loop
M2 helix
M2-M3 loop
Subunit A
Subunit B
Subunit C
Subunit D
Subunit E
V39
Y28
F37
E35
K33
L111
P113
L114
F116
R117
Y119
R118
I240
L241
I233
E222
P247
Y251

Supplementary Figure 6. Density validation of various regions in the *i3o2* state.

Density maps of key structural regions—β1–β2 loop, Pro-loop, M2 helix, and M2–M3 loop—are shown for subunits A through E in the *i3o2* state. Subunits in the *i* and *o* conformations are colored dodger blue and deep pink, respectively. Corresponding model segments are depicted as sticks overlaid with semi-transparent density maps, colored to match each conformation.

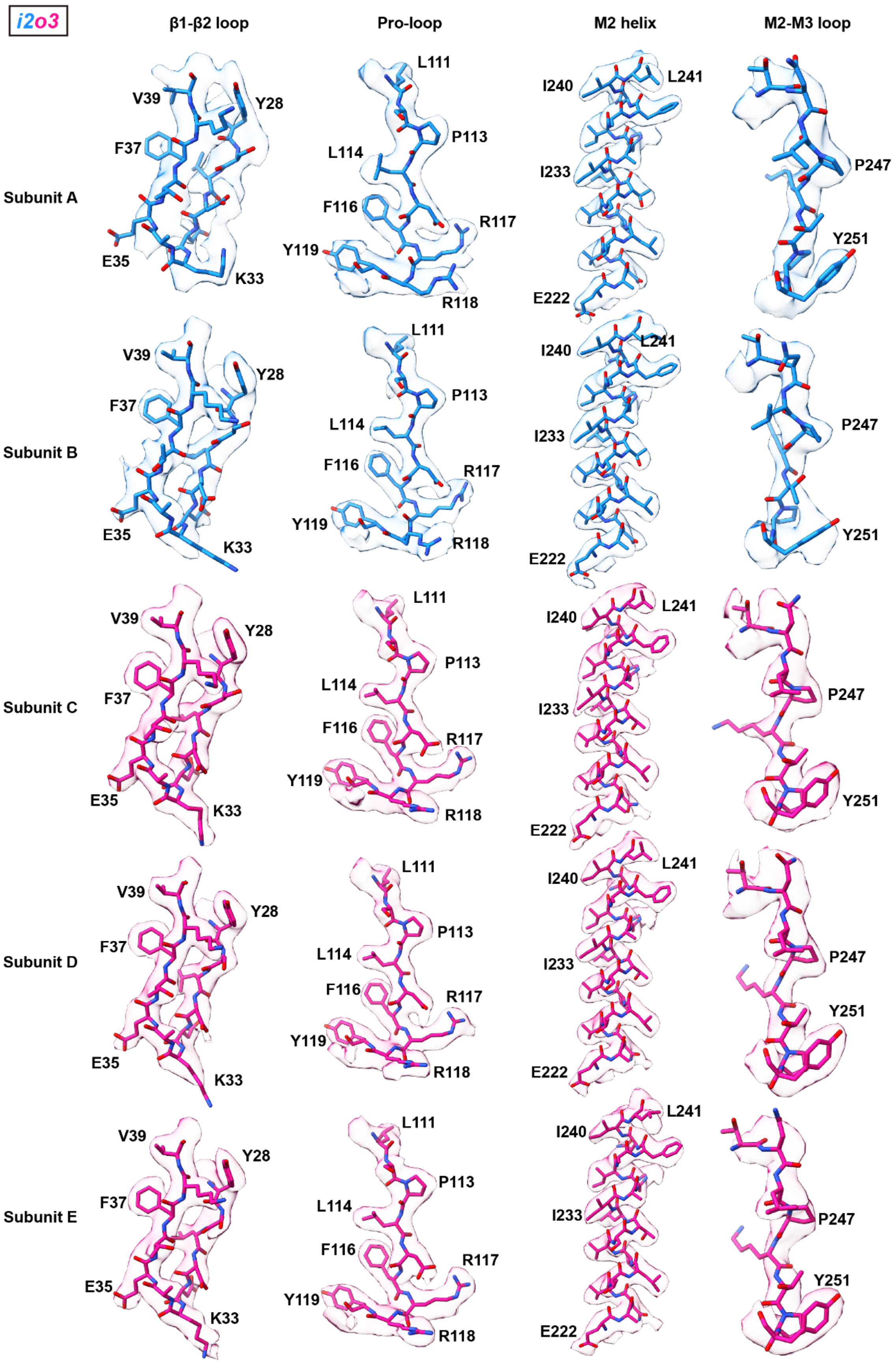
i2o3
β1-β2 loop
Pro-loop
M2 helix
M2-M3 loop
Subunit A
Subunit B
Subunit C
Subunit D
Subunit E
V39
Y28
F37
E35
K33
L111
P113
L114
F116
R117
Y119
R118
I240
L241
I233
E222
P247
Y251

Supplementary Figure 7. Density validation of various regions in the *i2o3* state.

Density maps of key structural regions—β1–β2 loop, Pro-loop, M2 helix, and M2–M3 loop—are shown for subunits A through E in the *i2o3* state. Subunits in the *i* and *o* conformations are colored dodger blue and deep pink, respectively. Corresponding model segments are depicted as sticks overlaid with semi-transparent density maps, colored to match each conformation.

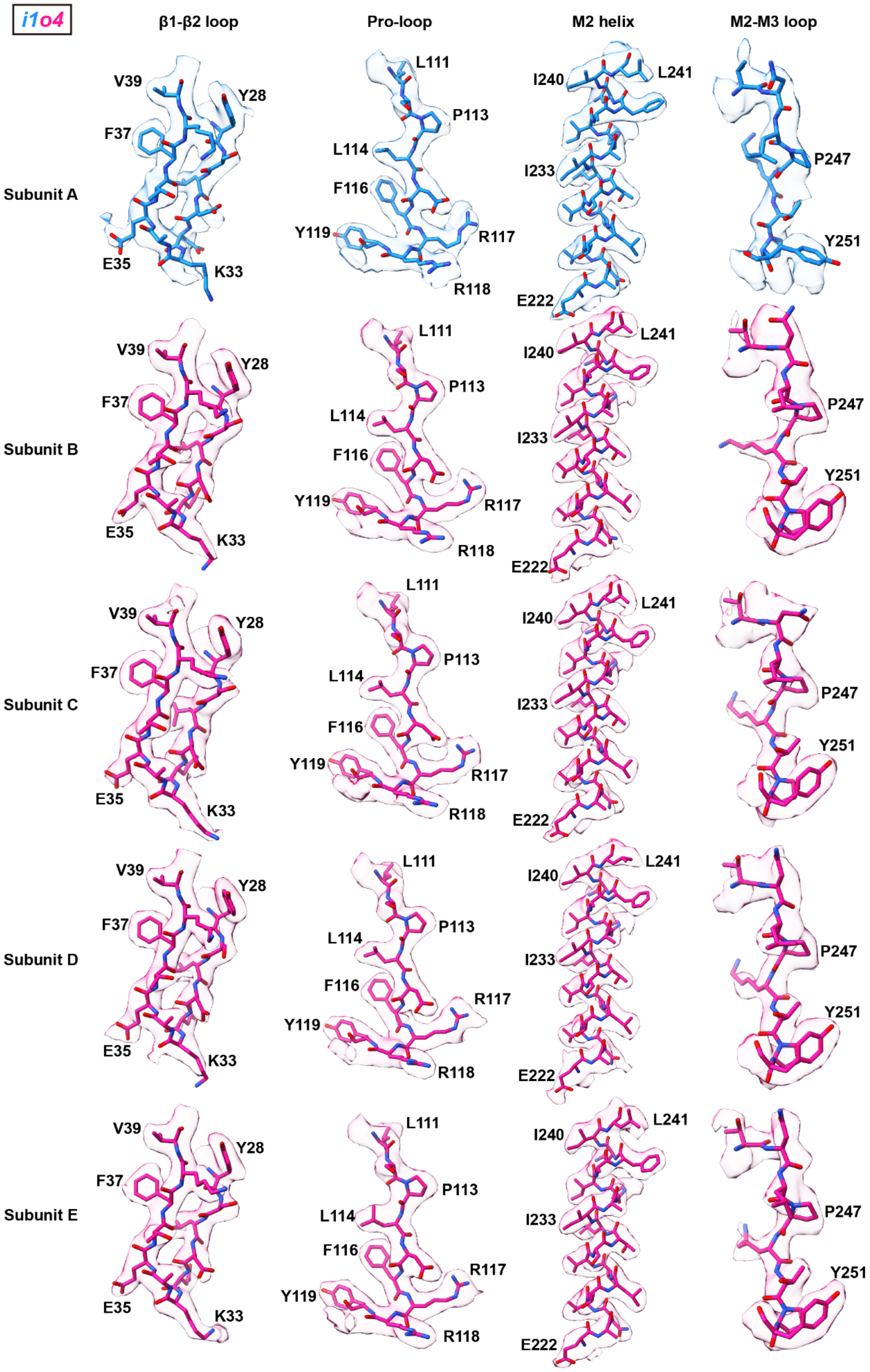
i1o4
β1-β2 loop
Pro-loop
M2 helix
M2-M3 loop
Subunit A
Subunit B
Subunit C
Subunit D
Subunit E
V39
Y28
F37
E35
K33
L111
P113
L114
F116
Y119
R117
R118
I240
L241
I233
E222
P247
Y251

Supplementary Figure 8. Density validation of various regions in the *i1o4* state.

Density maps of key structural regions—β1–β2 loop, Pro-loop, M2 helix, and M2–M3 loop—are shown for subunits A through E in the *i1o4* state. Subunits in the *i* and *o* conformations are colored dodger blue and deep pink, respectively. Corresponding model segments are depicted as sticks overlaid with semi-transparent density maps, colored to match each conformation.

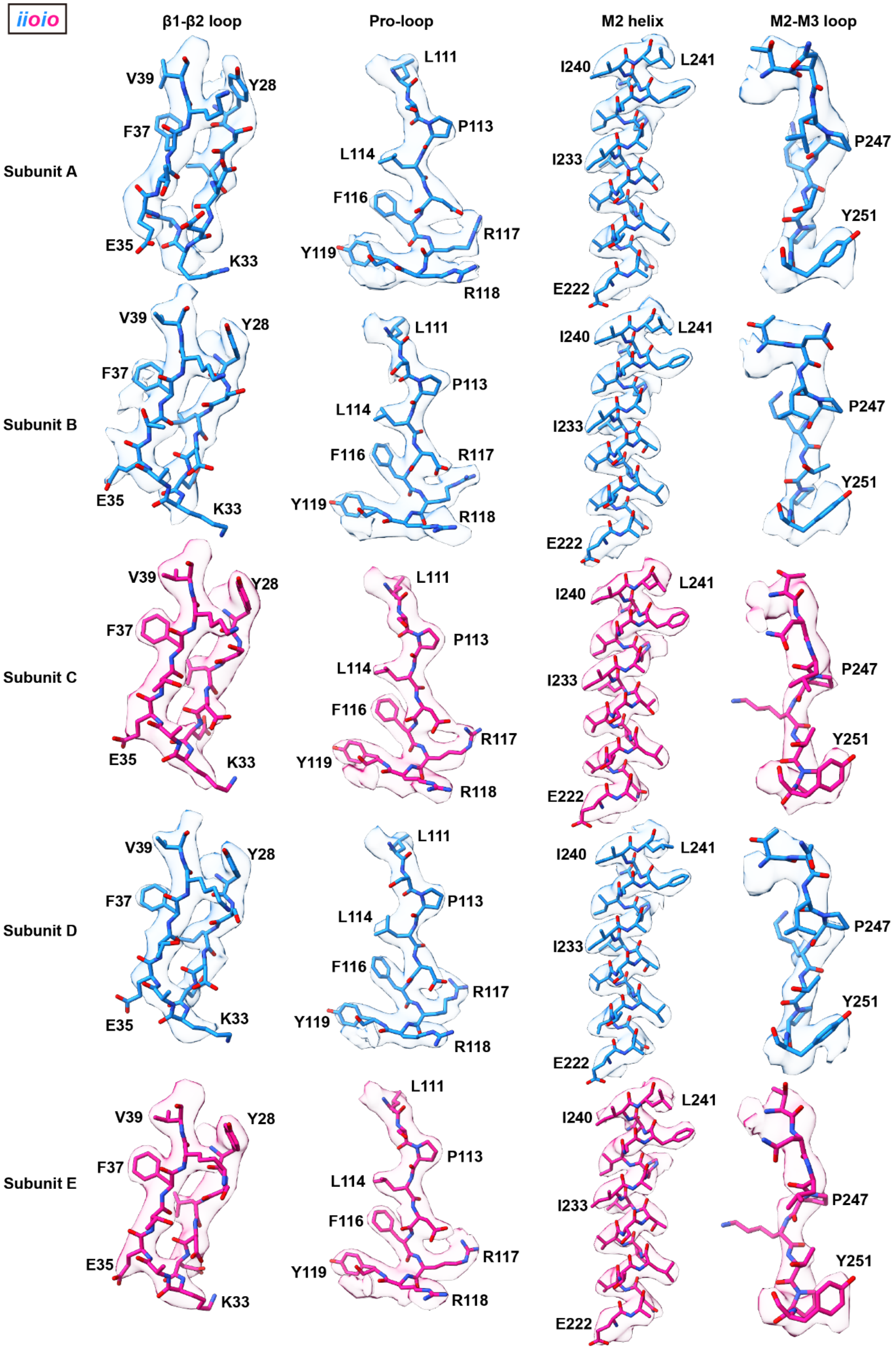
iioio
β1-β2 loop
Pro-loop
M2 helix
M2-M3 loop
Subunit A
Subunit B
Subunit C
Subunit D
Subunit E
V39
Y28
F37
E35
K33
L111
P113
L114
F116
R117
Y119
R118
I240
L241
I233
E222
P247
Y251

Supplementary Figure 9. Density validation of various regions in the *iioio* state. Density maps of key structural regions—β1–β2 loop, Pro-loop, M2 helix, and M2–M3 loop—are shown for subunits A through E in the *iioio* state. Subunits in the *i* and *o* conformations are colored dodger blue and deep pink, respectively. Corresponding model segments are depicted as sticks overlaid with semi-transparent density maps, colored to match each conformation.

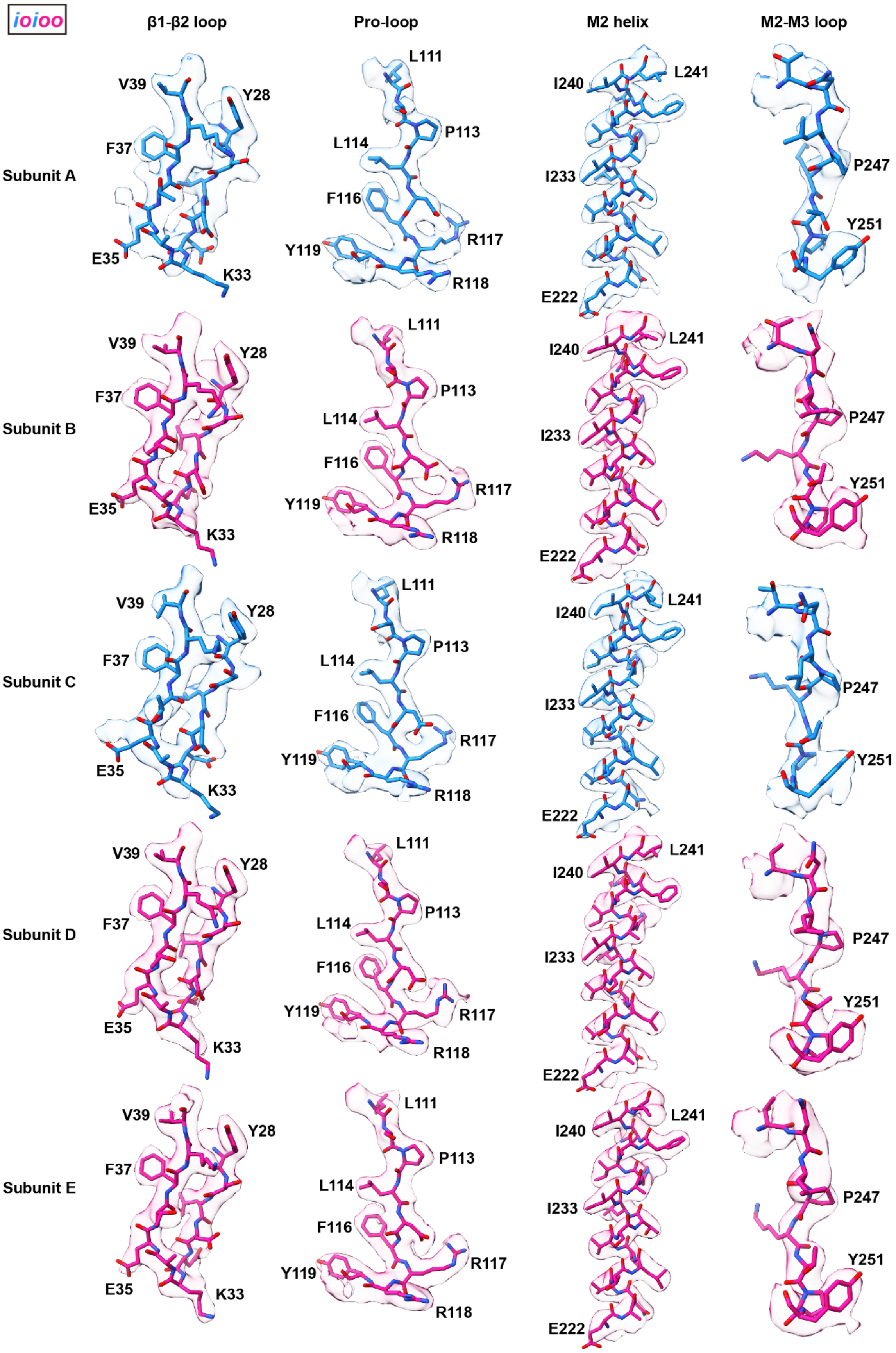
ioioo
β1-β2 loop
Pro-loop
M2 helix
M2-M3 loop
Subunit A
Subunit B
Subunit C
Subunit D
Subunit E
V39
Y28
F37
E35
K33
L111
P113
L114
F116
R117
Y119
R118
I240
L241
I233
E222
P247
Y251

Supplementary Figure 10. Density validation of various regions in the *ioioo* state. Density maps of key structural regions—β1–β2 loop, Pro-loop, M2 helix, and M2–M3 loop—are shown for subunits A through E in the *ioioo* state. Subunits in the *i* and *o* conformations are colored dodger blue and deep pink, respectively. Corresponding model segments are depicted as sticks overlaid with semi-transparent density maps, colored to match each conformation.

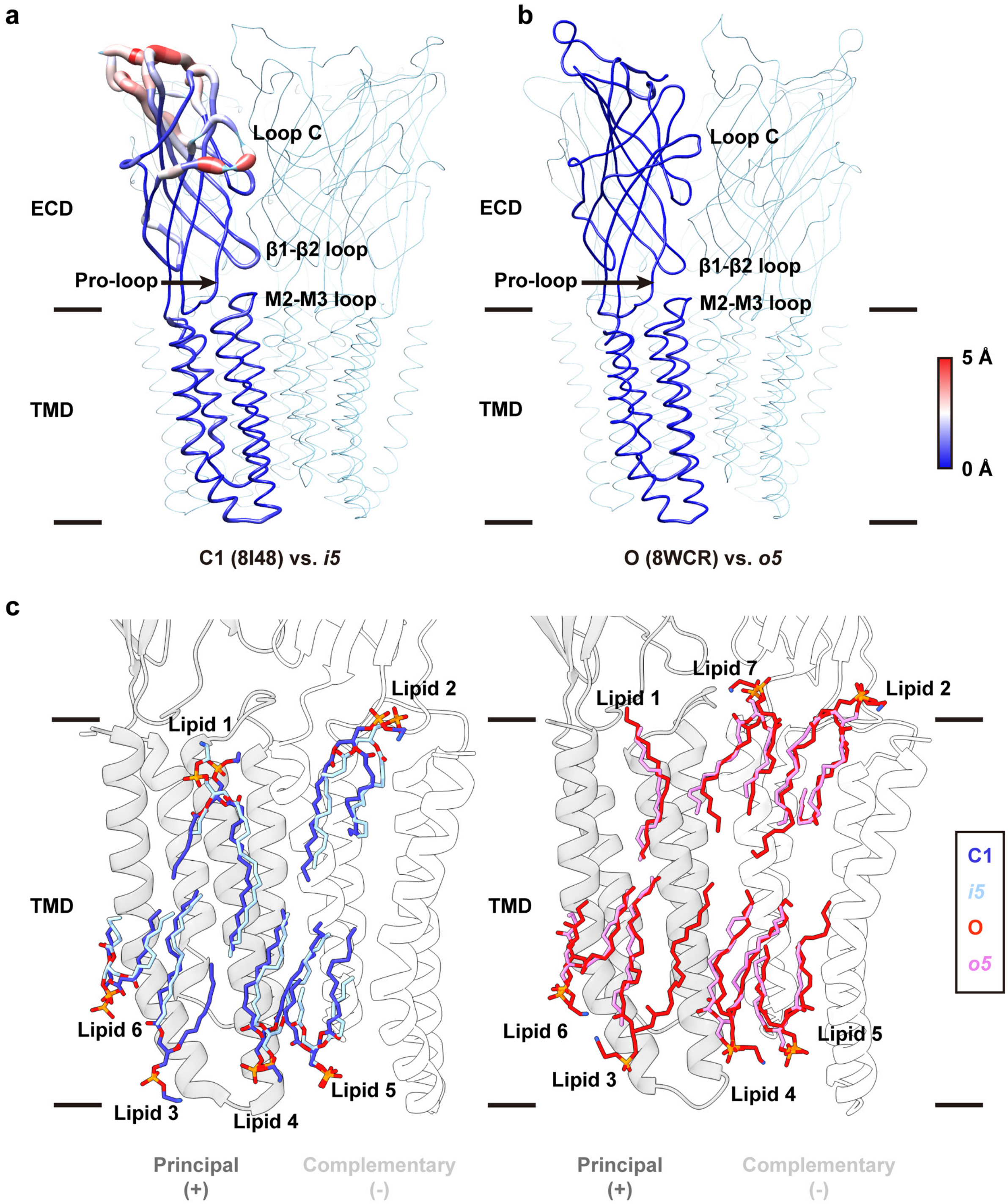

Supplementary Figure 11. Comparison of C1, *i5*, O and *o5* states. a Pairwise RMSD$_{C\alpha}$ comparison between the C1 and *i5* states. **b** Pairwise RMSD$_{C\alpha}$ comparison between the O and *o5* states. For clarity, one subunit is shown as a putty representation colored by RMSD$_{C\alpha}$, while other subunits are depicted in cyan licorice style. A color scale for RMSD$_{C\alpha}$ and a ribbon thickness key are included. **c** Superposition of C1 (blue) and *i5* (cyan) states,

and the O (red) and *o5* (pink) states. For clarity, only the principal (dark grey) and complementary subunits (light grey) are shown, and lipids are displayed as sticks.

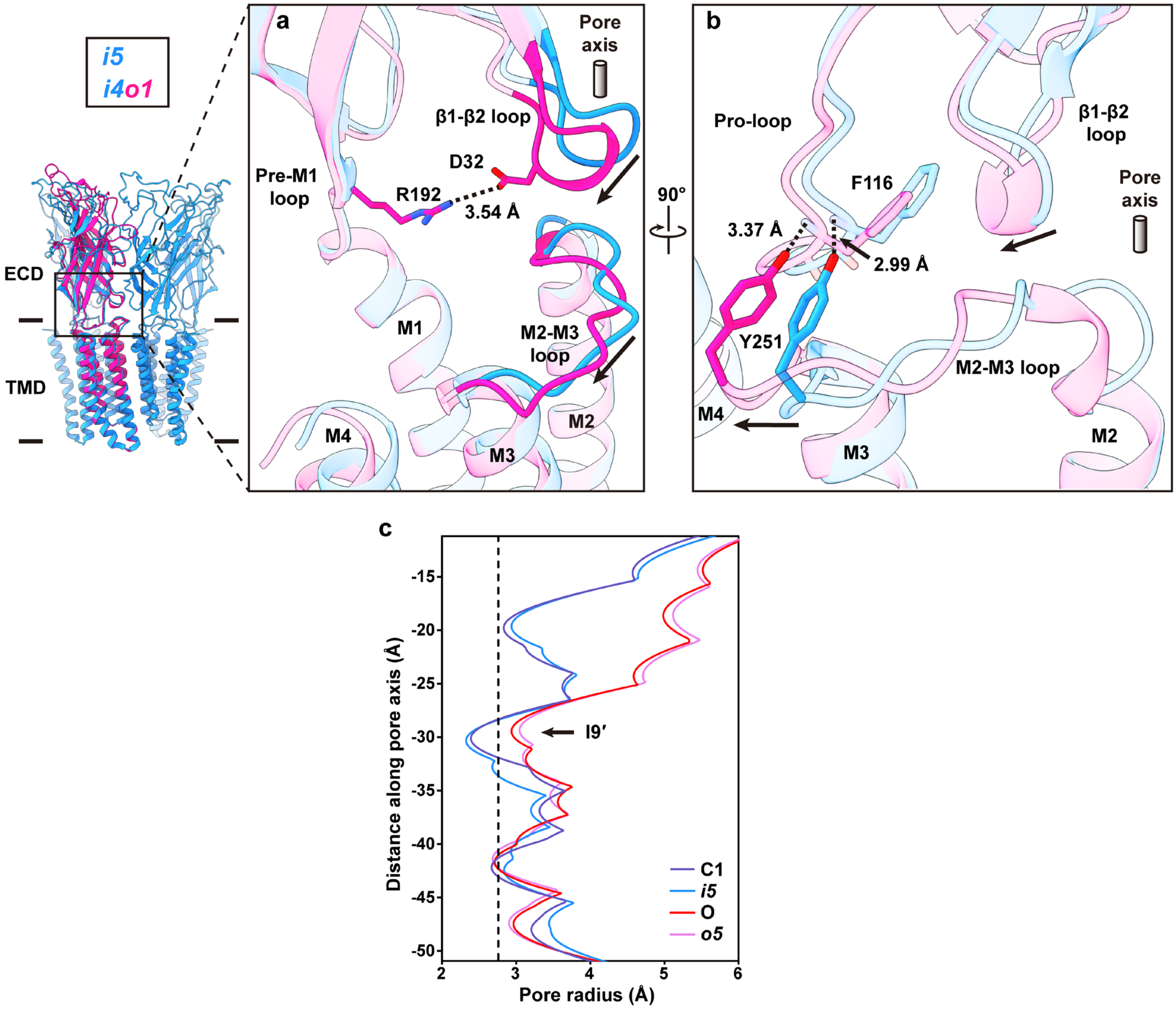

Supplementary Figure 12. GLIC activation and conformational changes at the domain interface. Superposition of the *i5* (dodger blue) and *i4o1* (deep pink) states shown as cartoons. The zoomed-in view highlights a downward displacement of the β1–β2 loop in the *i4o1* state. The hydrogen bonds between (**a**) D32 and R192 and (**b**) F116 and Y251 are shown. D32, R192, F116 and Y251 are shown as sticks. **c** Pore radius comparison among C1, *i5*, O, and *o5* states. The black dashed line indicates the approximate radius of a hydrated Na^{+} ion. The position of I9' is indicated by arrow.

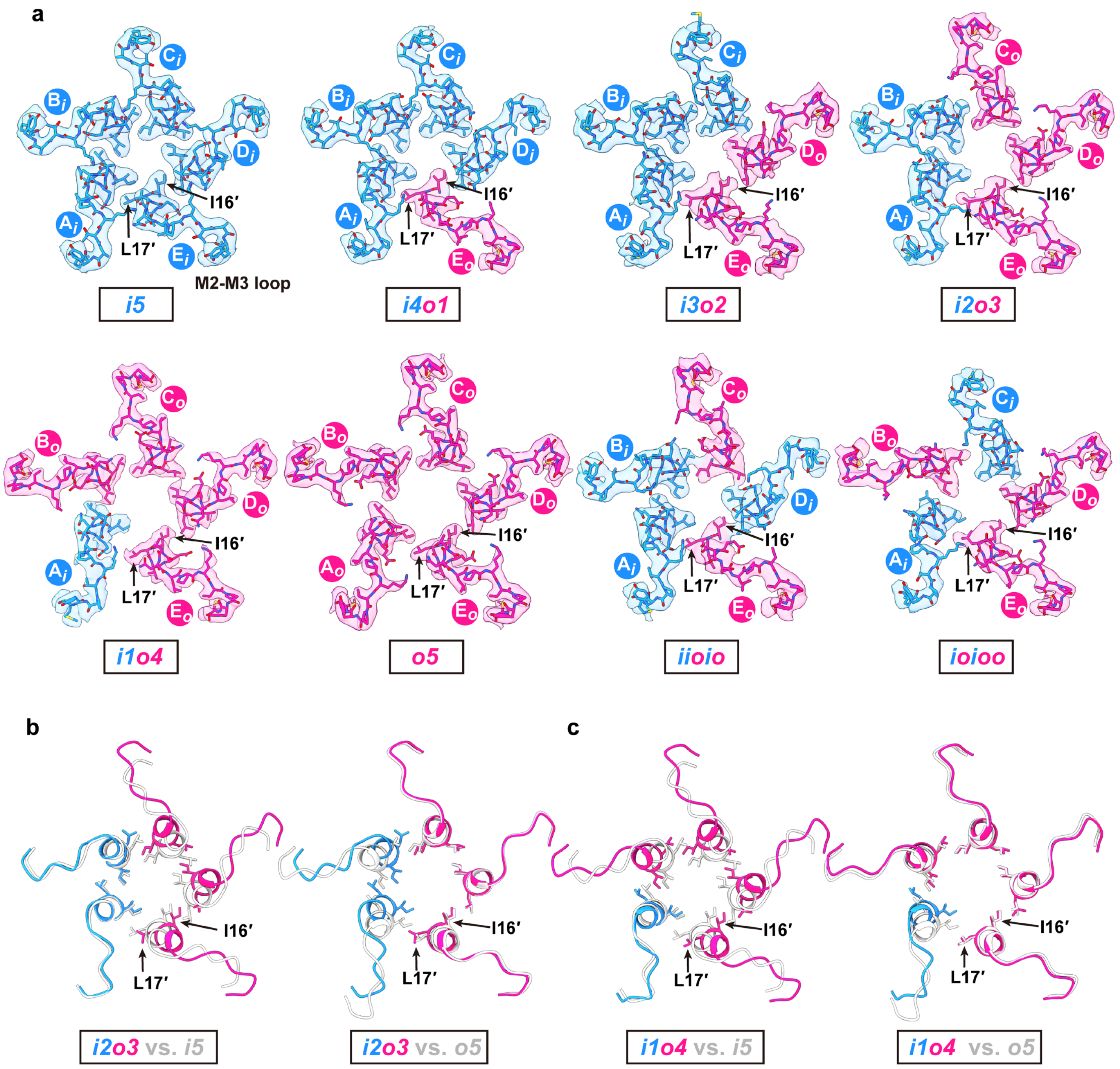

Supplementary Figure 13. Density validation and comparison of M2 helix and M2–M3 loops. a Density maps of the pore-lining M2 helix and M2–M3 loops are shown for all subunits in the *i5, i4o1, i3o2, i2o3, i1o4, o5, iioio,* and *ioioo* states. Model segments are depicted as sticks overlaid with semi-transparent density maps. **b** Structural comparisons of *i2o3* with *i5* (grey; left) and *o5* (grey; right). **c** Structural comparisons of *i1o4* with *i5*

(grey; left) and *o5* (grey; right). I16' and L17' are shown as sticks. Subunits in the *i* and *o* conformations are colored dodger blue and deep pink, respectively.

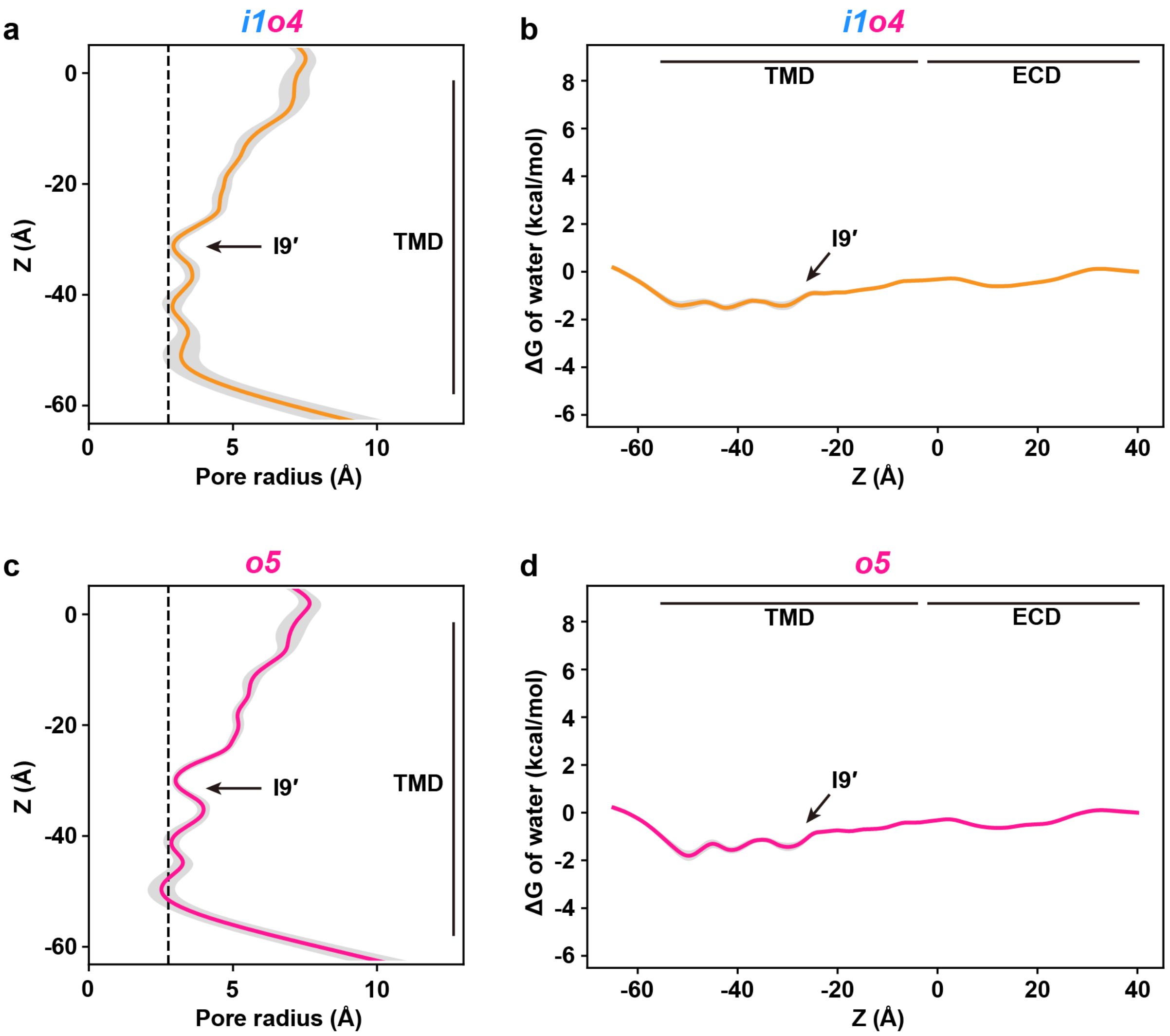

Supplementary Figure 14. Molecular dynamics simulations of *i1o4* and *o5* states. a Mean pore radius profiles for *i1o4* (orange) with the corresponding standard deviation shown in grey. **b** Free-energy profiles for a water molecule along the central pore axis in the *i1o4* state. **c** Mean pore radius profiles for *o5* (deep pink) with the corresponding standard deviation shown in grey. **d** Free-energy profiles for a water molecule along the central pore axis in the *o5* state. The black dashed line indicates the approximate diameter of a single water molecule . The position of I9' is marked for reference.

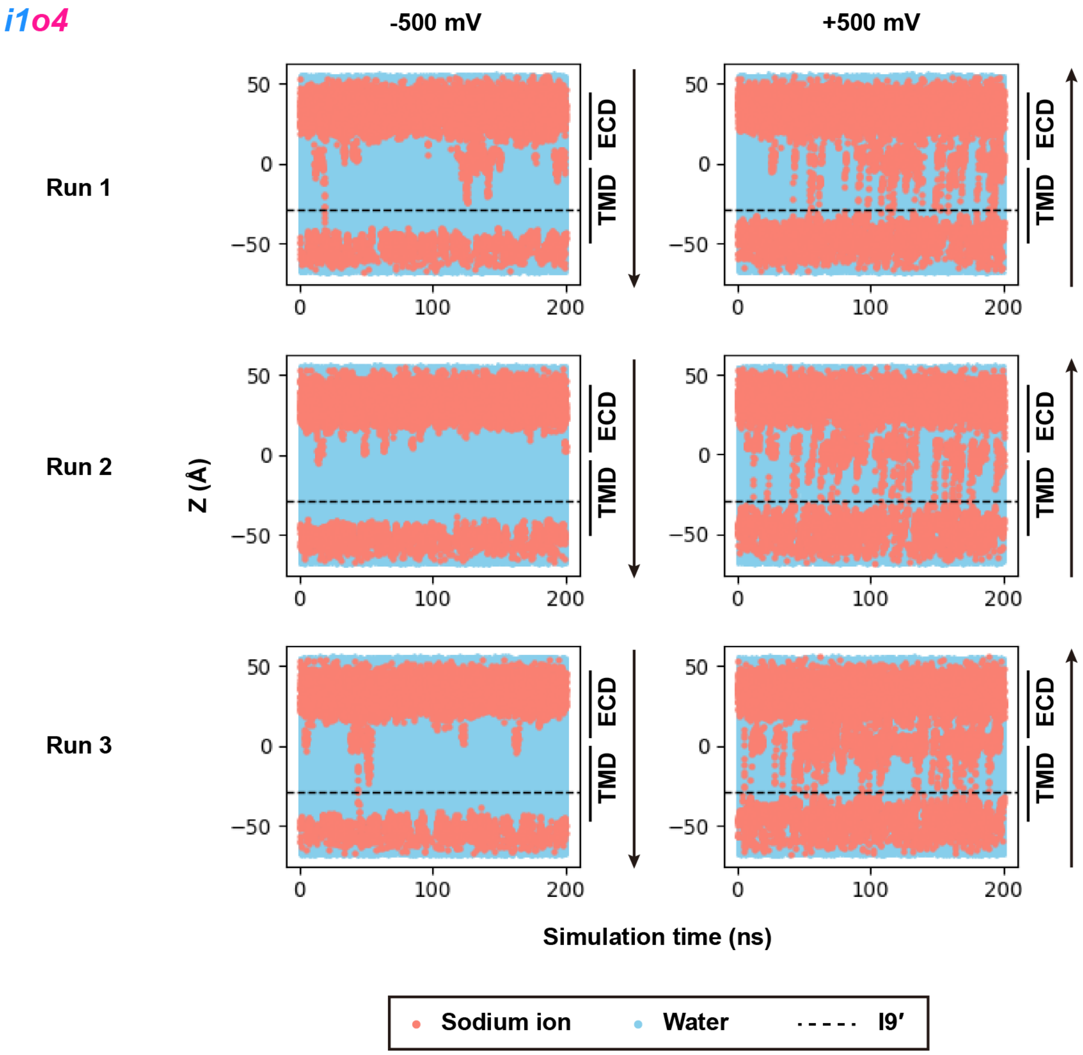

Supplementary Figure 15. Ion permeation MD simulations of *i1o4* state performed in the presence of transmembrane potentials. Results for three repeats with -500 mV or +500 mV external electric fields are shown. The sodium ions and water molecules are colored in salmon and blue, respectively. The positions of ECD and TMD are indicated on the right of each plot. The position of I9′ is indicated by a dashed line. The vertical arrows show the directions of ion passage.

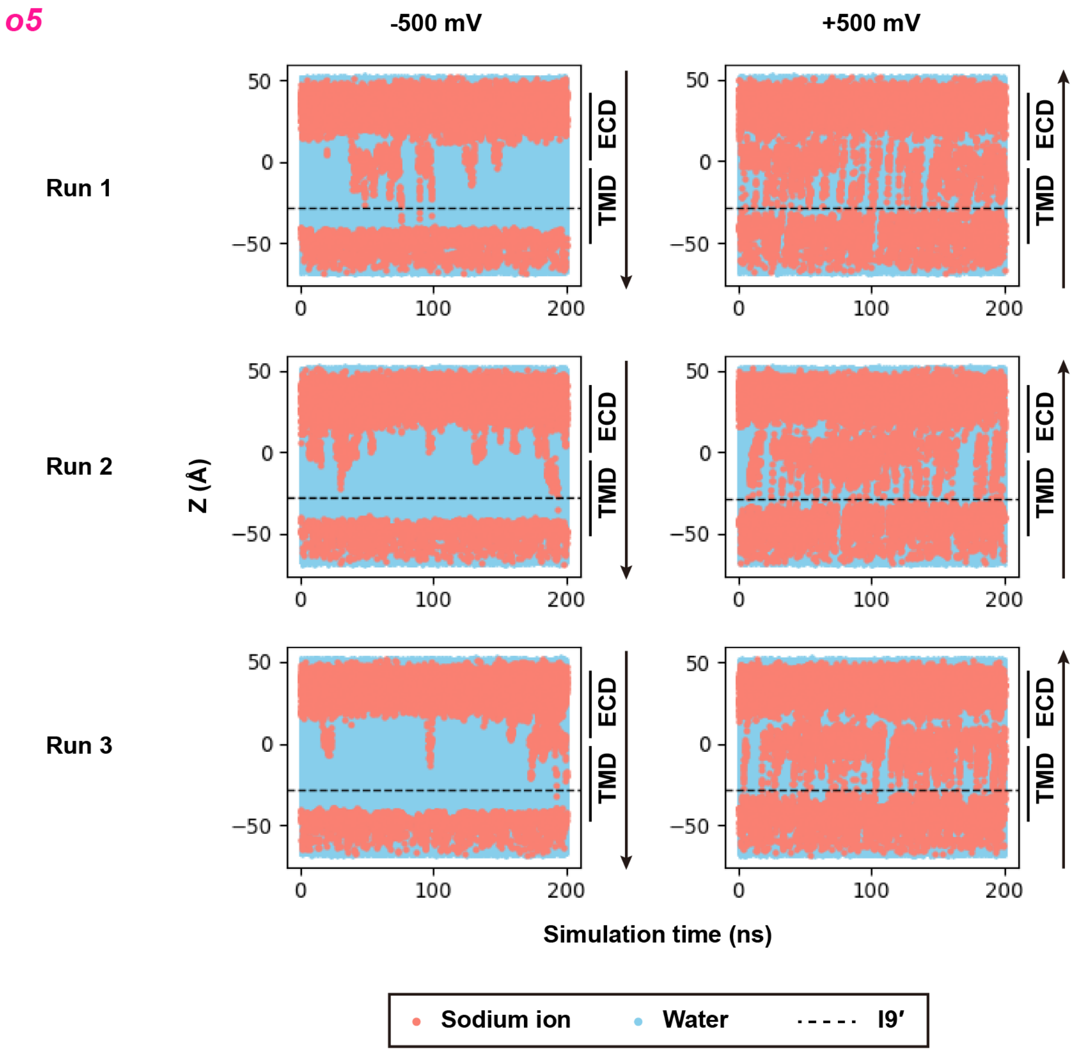

Supplementary Figure 16. Ion permeation MD simulations of *o5* state performed in the presence of transmembrane potentials. Results for three repeats with -500 mV or +500 mV external electric fields are shown. The sodium ions and water molecules are colored in salmon and blue, respectively. The positions of ECD and TMD are indicated on the right of each plot. The position of I9′ is indicated by a dashed line. The vertical arrows show the directions of ion passage.

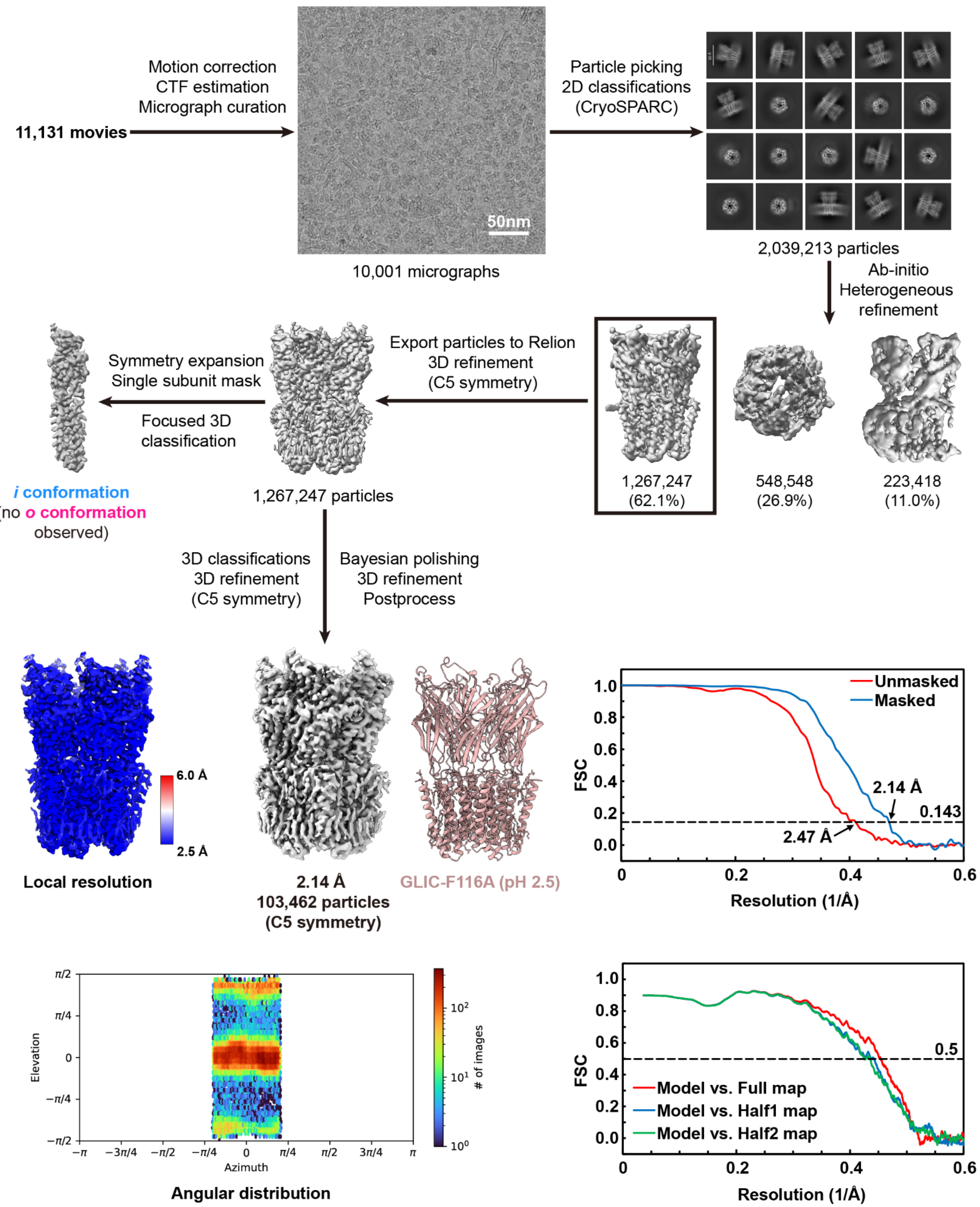
11,131 movies
Motion correction
CTF estimation
Micrograph curation
50nm
10,001 micrographs
Particle picking
2D classifications
(CryoSPARC)
2,039,213 particles
Ab-initio
Heterogeneous
refinement
1,267,247
(62.1%)
548,548
(26.9%)
223,418
(11.0%)
Export particles to Relion
3D refinement
(C5 symmetry)
1,267,247 particles
Symmetry expansion
Single subunit mask
Focused 3D
classification
i conformation
(no o conformation
observed)
3D classifications
3D refinement
(C5 symmetry)
Bayesian polishing
3D refinement
Postprocess
6.0 Å
2.5 Å
Local resolution
2.14 Å
103,462 particles
(C5 symmetry)
GLIC-F116A (pH 2.5)
Unmasked
Masked
FSC
2.14 Å
0.143
2.47 Å
Resolution (1/Å)
Elevation
Azimuth
# of images
Angular distribution
0.5
Model vs. Full map
Model vs. Half1 map
Model vs. Half2 map
FSC
Resolution (1/Å)

Supplementary Figure 17. Cryo-EM data processing workflow for the GLIC-F116A mutant. Cryo-EM data processing for the GLIC-F116A mutant includes a representative micrograph, 2D class averages, ab-initio reconstruction, heterogeneous refinement, and non-uniform refinement. Particles were subjected to symmetry expansion and focused classification, which revealed that all particles correspond to the *i5* state. The final 3D reconstruction using C5 symmetry achieved a nominal resolution of 2.14 Å. Gold-standard Fourier Shell Correlation (FSC), angular distribution, local resolution estimation, and model-to-map validation FSC curves are shown. FSC curves before (red) and after (blue) masking are included, with the dashed line indicating the 0.143 cutoff. Angular distributions were computed using cryoSPARC. The reconstructed map is colored by local resolution, estimated using ResMap, with a resolution color key provided.

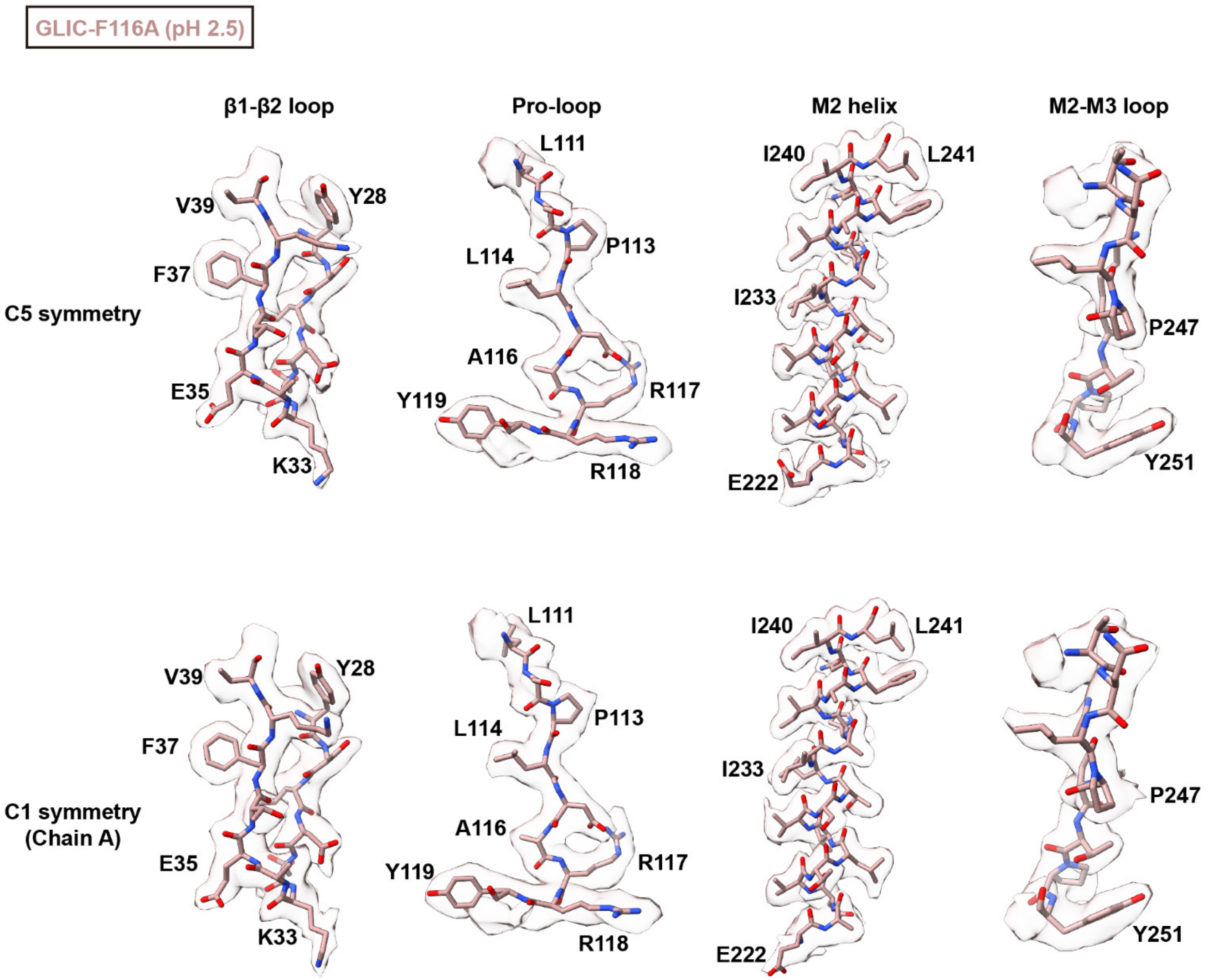

Supplementary Figure 18. Density validation of various regions in the GLIC-F116A mutant. Density maps of key structural regions—β1–β2 loop, Pro-loop, M2 helix, and M2– M3 loop—are shown for GLIC-F116A mutant. Corresponding model segments are depicted as sticks overlaid with semi-transparent density maps, colored to match each conformation.

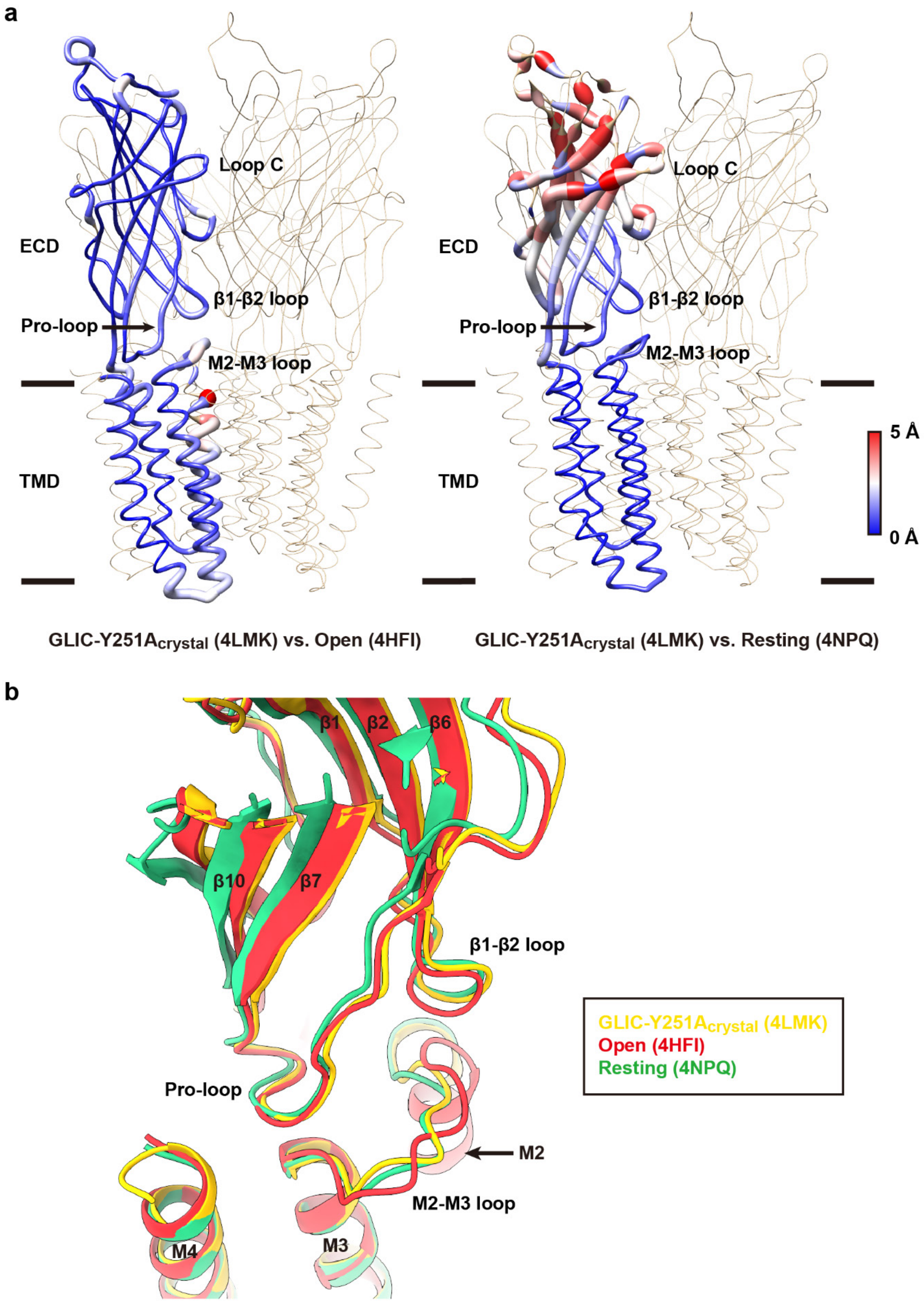
a
Loop C
ECD
β1-β2 loop
Pro-loop
M2-M3 loop
TMD
Loop C
ECD
β1-β2 loop
Pro-loop
M2-M3 loop
TMD
5 Å
0 Å
GLIC-Y251A$_{crystal}$ (4LMK) vs. Open (4HFI)
GLIC-Y251A$_{crystal}$ (4LMK) vs. Resting (4NPQ)
b
β1
β2
β6
β10
β7
β1-β2 loop
Pro-loop
M2
M2-M3 loop
M4
M3
GLIC-Y251A$_{crystal}$ (4LMK)
Open (4HFI)
Resting (4NPQ)

Supplementary Figure 19. Comparison of the GLIC-Y251A crystal structure with open and resting states. a Pairwise $RMSD_{C\alpha}$ comparison between the crystal structure of GLIC-Y251A (PDB: 4LMK) and the open state (PDB: 4HFI). Pairwise $RMSD_{C\alpha}$ comparison between GLIC-Y251A (PDB: 4LMK) and the resting state (PDB: 4NPQ). For clarity, one subunit is shown as a putty representation colored by $RMSD_{C\alpha}$, and the remaining subunits are displayed in tan using a licorice style. A color scale and ribbon thickness key are provided. **b** Superposition of GLIC-Y251A crystal structure (PDB: 4LMK; yellow) with open (PDB: 4HFI; red) and resting states (PDB: 4NPQ; green) are shown in cartoon.

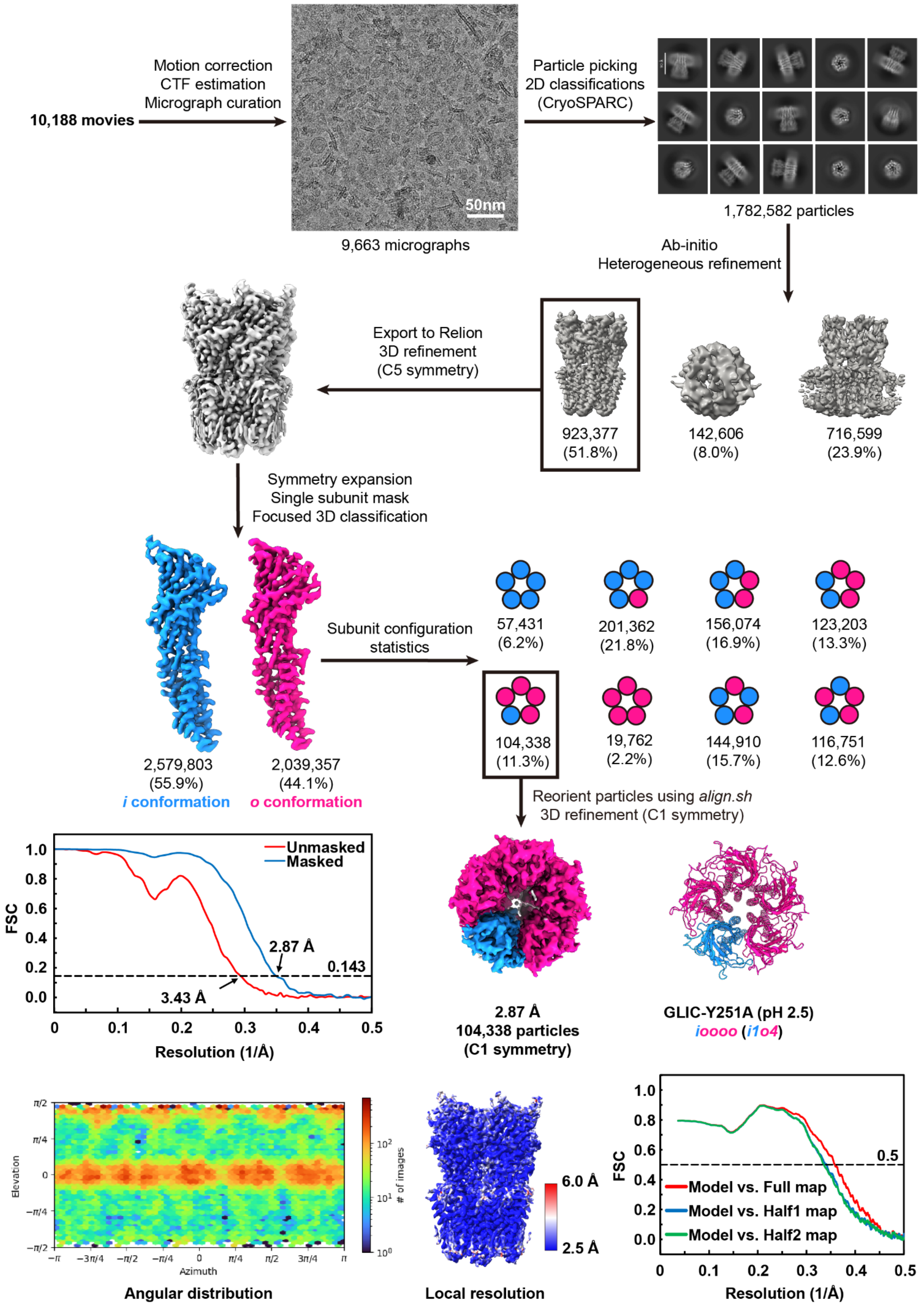

10,188 movies
Motion correction
CTF estimation
Micrograph curation
50nm
9,663 micrographs
Particle picking
2D classifications
(CryoSPARC)
1,782,582 particles
Ab-initio
Heterogeneous refinement
923,377
(51.8%)
142,606
(8.0%)
716,599
(23.9%)
Export to Relion
3D refinement
(C5 symmetry)
Symmetry expansion
Single subunit mask
Focused 3D classification
2,579,803
(55.9%)
i conformation
2,039,357
(44.1%)
o conformation
Subunit configuration
statistics
57,431
(6.2%)
201,362
(21.8%)
156,074
(16.9%)
123,203
(13.3%)
104,338
(11.3%)
19,762
(2.2%)
144,910
(15.7%)
116,751
(12.6%)
Reorient particles using align.sh
3D refinement (C1 symmetry)
Unmasked
Masked
FSC
2.87 Å
0.143
3.43 Å
Resolution (1/Å)
2.87 Å
104,338 particles
(C1 symmetry)
GLIC-Y251A (pH 2.5)
ioooo (i1o4)
Elevation
Azimuth
# of images
Angular distribution
6.0 Å
2.5 Å
Local resolution
0.5
Model vs. Full map
Model vs. Half1 map
Model vs. Half2 map
Resolution (1/Å)

Supplementary Figure 20. Cryo-EM data processing workflow for the GLIC-Y251A mutant. Cryo-EM data processing for the GLIC-Y251A mutant includes a representative micrograph, 2D class averages, ab-initio reconstruction, heterogeneous refinement, and non-uniform refinement. Particles were subjected to symmetry expansion and focused classification. The final 3D reconstruction for *i1o4* state achieved a nominal resolution of 2.87 Å. Gold-standard Fourier Shell Correlation (FSC), angular distribution, local resolution estimation, and model-to-map validation FSC curves are shown. FSC curves before (red) and after (blue) masking are included, with the dashed line indicating the 0.143 cutoff. Angular distributions were computed using cryoSPARC. Reconstructed map is colored by local resolution, estimated using ResMap, with a resolution color key provided.

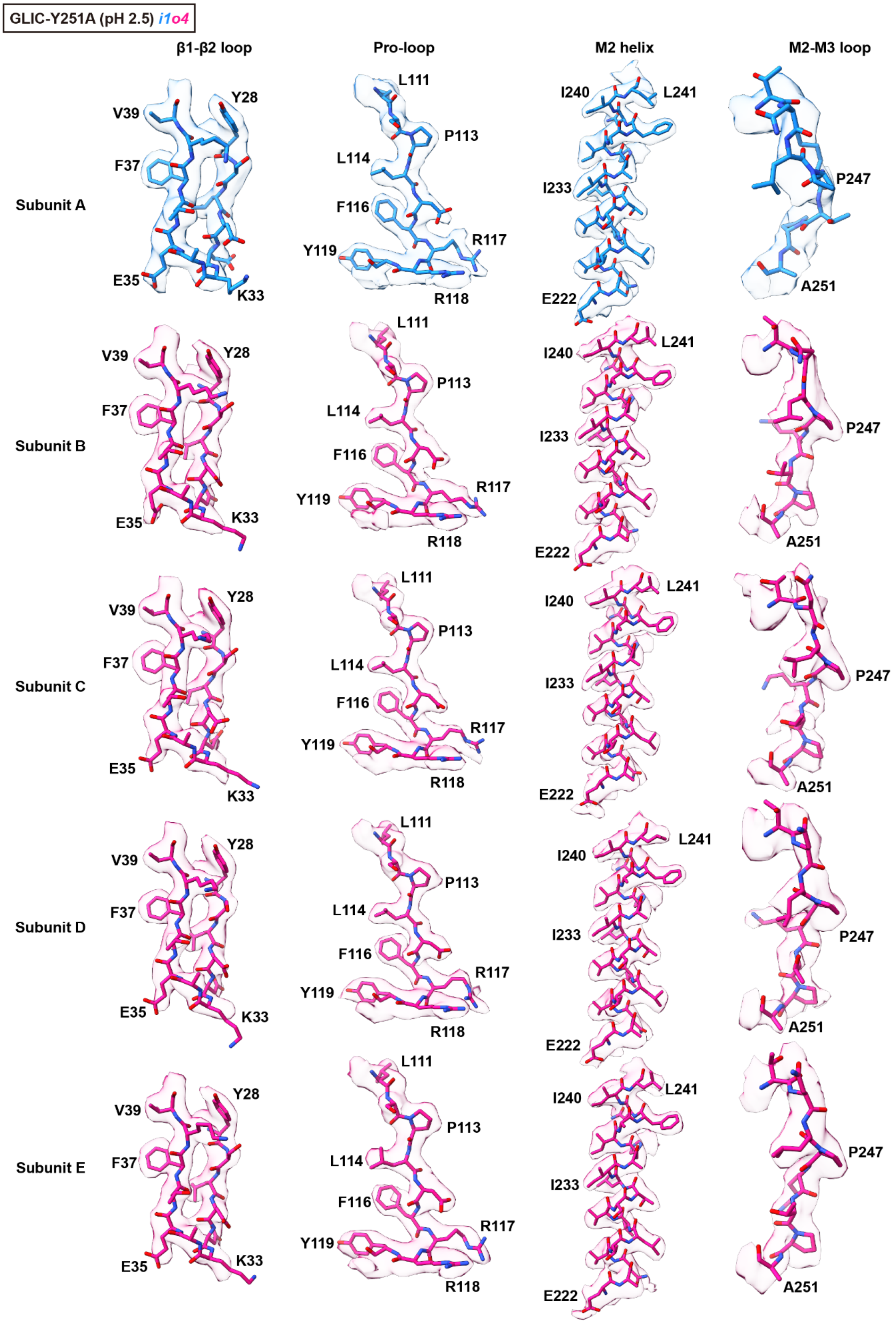
GLIC-Y251A (pH 2.5) i1o4
β1-β2 loop
Pro-loop
M2 helix
M2-M3 loop
Subunit A
Subunit B
Subunit C
Subunit D
Subunit E
V39
Y28
F37
E35
K33
L111
P113
L114
F116
R117
Y119
R118
I240
L241
I233
E222
P247
A251

Supplementary Figure 21. Density validation of various regions in the GLIC-Y251A mutant. Density maps of key structural regions—β1–β2 loop, Pro-loop, M2 helix, and M2– M3 loop—are shown for GLIC-Y251A mutant. Subunits in the *i* and *o* conformations are colored dodger blue and deep pink, respectively. Corresponding model segments are depicted as sticks overlaid with semi-transparent density maps, colored to match each conformation.

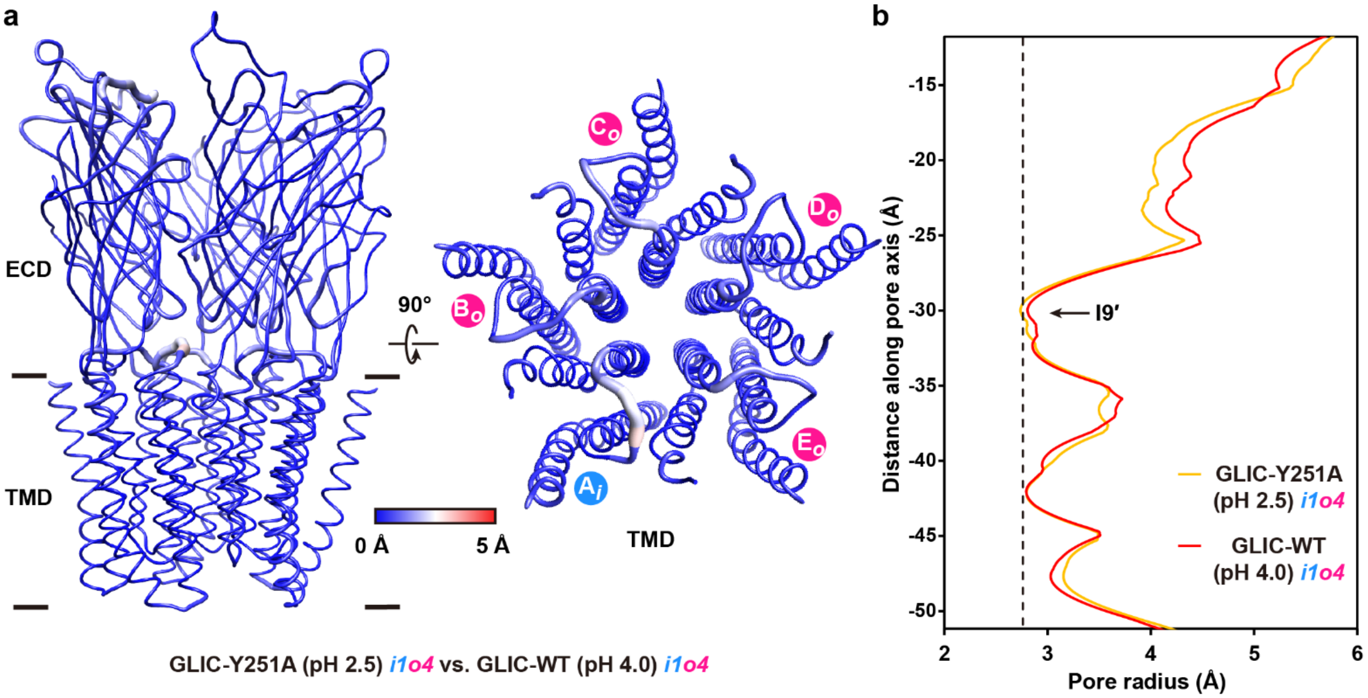

Supplementary Figure 22. Comparison between *i1o4* states of GLIC-Y251A at pH 2.5 and *i1o4* states of GLIC-WT at pH 4.0. a Pairwise $RMSD_{C\alpha}$ comparison between the *i1o4* state of the Y251A mutant at pH 2.5 and *i1o4* state of GLIC-WT at pH 4.0, shown in side view and top view (sliced at TMD). Model is shown in licorice representation and colored by $RMSD_{C\alpha}$. A color scale and ribbon thickness key are included. **b** Pore radius profiles of *i1o4* states are plotted along the central pore axis for both GLIC-Y251A at pH 2.5 and GLIC-WT at pH 4.0. The black dashed line indicates the approximate radius of a hydrated Na^+ ion.

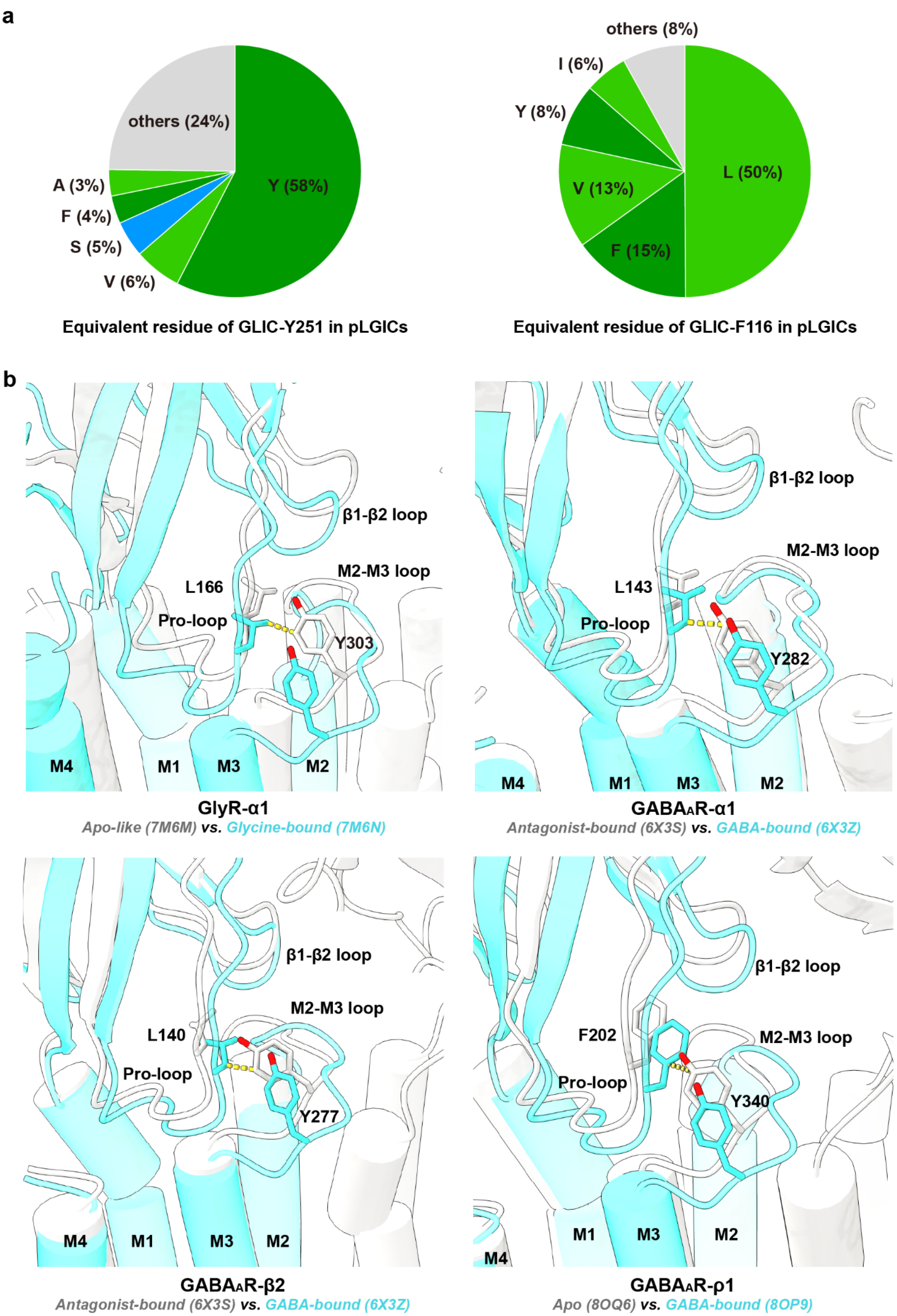
a
others (24%)
Y (58%)
A (3%)
F (4%)
S (5%)
V (6%)
Equivalent residue of GLIC-Y251 in pLGICs
others (8%)
I (6%)
Y (8%)
V (13%)
F (15%)
L (50%)
Equivalent residue of GLIC-F116 in pLGICs
b
β1-β2 loop
M2-M3 loop
L166
Pro-loop
Y303
M4
M1
M3
M2
GlyR-α1
Apo-like (7M6M) vs. Glycine-bound (7M6N)
β1-β2 loop
M2-M3 loop
L143
Pro-loop
Y282
M4
M1
M3
M2
GABAAR-α1
Antagonist-bound (6X3S) vs. GABA-bound (6X3Z)
β1-β2 loop
M2-M3 loop
L140
Pro-loop
Y277
M4
M1
M3
M2
GABAAR-β2
Antagonist-bound (6X3S) vs. GABA-bound (6X3Z)
β1-β2 loop
F202
M2-M3 loop
Pro-loop
Y340
M1
M3
M2
M4
GABAAR-ρ1
Apo (8OQ6) vs. GABA-bound (8OP9)

Supplementary Figure 23. The residue conservation in Pro-loop and M2-M3 loop in the pLGICs family. a Amino acids conservation at Y251 and F116 equivalent among pLGICs are shown in pie charts. **b** Superposition of apo (grey) and glycine bound (cyan) GlyR-α1(top left); antagonist bound (grey) and GABA bound (cyan) $GABA_AR$-α1 (top right); antagonist bound (grey) and GABA bound (cyan) $GABA_AR$-β2 (bottom left); apo (grey) and GABA bound (cyan) $GABA_AR$-ρ1 (bottom right) are shown in cartoon. L166, Y303, L143, Y282, L140, Y277, F202, Y340 are shown as sticks. A representative clash is shown as dashed line (yellow).

Supplementary Table 1. Comprehensive summary of cryo-EM parameters for data acquisition, image processing, 3D reconstruction, atomic model refinement, and validation. Data collection parameters include microscope magnification, accelerating voltage, energy filter slit width, total electron exposure, defocus range, and calibrated pixel size. Reconstruction metrics include the number of micrographs and particles used, imposed symmetry, final map resolution based on the 0.143 Fourier Shell Correlation (FSC) criterion, and map sharpening B factor. Refinement statistics list the initial atomic model (PDB code), the number of non-hydrogen atoms, protein residues, ligands, model-to-map correlation (CC mask), and average B factors. Geometric quality is assessed through root-mean-square (RMS) deviations in bond lengths and angles. Model validation includes MolProbity score, clashscore, percentage of poor rotamers, and Ramachandran plot statistics (favored, allowed, and disallowed regions).

	GLIC-WT (pH 4.0)								GLIC-F116A (pH 2.5)	GLIC-Y251A (pH 2.5) *ioooo*
	iiiii EMDB-62927 PDB-9LAG	*iiiio* EMDB-62929 PDB-9LAI	*iiioo* EMDB-62930 PDB-9LAJ	*iiooo* EMDB-62931 PDB-9LAK	*ioooo* EMDB-62937 PDB-9LB9	*ooooo* EMDB-62938 PDB-9LBA	*iioio* EMDB-62939 PDB-9LBB	*ioioo* EMDB-62940 PDB-9LBC	EMDB-62941 PDB-9LBD	EMDB-62942 PDB-9LBE
**Data Collection and processing**										
Magnification	165,000x								165,000x	165,000x
Voltage (kV)	300								300	300
Image filter	Selectris X								Selectris X	Selectris X
Slit width (eV)	10								10	10
Electron exposure ($e^-/Å^2$)	71								70	70
Defocus range (μm)	0.6 to 1.4								0.5 to 1.5	0.5 to 1.5
Pixel size (Å)	0.76								0.76	0.76
Micrographs	14,076								11,131	10,188
Final particle images (no.)	16,466	34,185	21,677	16,336	17,448	19,034	12,143	7,540	103,462	104,338
Symmetry imposed	C5	C1	C1	C1	C1	C5	C1	C1	C5	C1
Map resolution at 0.143 FSC (Å)	2.96	3.08	3.16	3.21	3.16	2.85	3.45	3.40	2.14	2.87
Map sharpening *B* factor ($Å^2$)	-15	-15	-15	-15	-15	-15	-15	-15	-8	-15
**Refinement**										
Initial model used	8I48	8I48	8I48	8WCR	8WCR	8WCR	8I48	8WCR	8WCR	8WCR

(PDB code)										
Model composition										
Non-hydrogen atoms	13,535	13,400	13,356	13,191	13,344	13,795	13,002	12,830	13,370	13,284
Protein residues	1,560	1,560	1,560	1,560	1,560	1,560	1,560	1,560	1,560	1,560
Ligands	30	29	28	22	27	40	10	5	30	26
CC mask	0.86	0.88	0.88	0.88	0.90	0.90	0.87	0.87	0.89	0.88
B factor (Å^2)										
Protein residues	106.92	97.99	86.94	83.37	62.44	68.94	85.43	75.15	72.63	79.28
Ligands	99.10	91.76	86.76	84.86	75.24	99.31	86.59	70.99	87.13	89.00
R.M.S. deviations										
Bond lengths (Å)	0.002	0.003	0.002	0.002	0.003	0.004	0.003	0.003	0.002	0.003
Bond angles (°)	0.510	0.514	0.487	0.479	0.488	0.822	0.563	0.532	0.498	0.462
Validation										
MolProbity score	1.25	1.59	1.35	1.32	1.33	1.35	1.61	1.47	1.16	1.22
Clashscore	3.03	4.13	3.97	3.83	3.70	4.04	5.31	4.15	3.14	2.67
Poor rotamers (%)	0.36	1.21	0.64	0.43	0.00	0.00	0.00	0.50	0.00	0.36
Ramachandran plot										
Favored (%)	97.10	95.23	97.03	97.16	97.03	97.10	95.35	96.00	97.74	97.03
Allowed (%)	2.90	4.77	2.97	2.84	2.97	2.90	4.65	4.00	2.26	2.97
Disallowed (%)	0	0	0	0	0	0	0	0	0	0

Supplementary Table 2. Comprehensive summary of cryo-EM parameters for data acquisition, image processing, 3D reconstruction, atomic model refinement, and validation of i5, o5, and GLIC-F116A at pH 2.5 in C1 symmetry. Data collection parameters and reconstruction metrics are shown in consistency with Supplementary Table 1.

	GLIC-WT (pH 4.0) *iiiii*	GLIC-WT (pH 4.0) *ooooo*	GLIC-F116A (pH 2.5)
**Data Collection and processing**			
Magnification	165,000x		165,000x
Voltage (kV)	300		300
Image filter	Selectris X		Selectris X
Slit width (eV)	10		10
Electron exposure (e^-/Å^2)	71		70
Defocus range (µm)	0.6 to 1.4		0.5 to 1.5
Pixel size (Å)	0.76		0.76
Micrographs	14,076		11,131
Final particle images (no.)	16,466	34,185	21,677
Symmtry imposed	C1	C1	C1
Map resolution at 0.143 FSC (Å)	3.30	3.08	2.95
Map sharpening *B* factor (Å^2)	-15	-15	-15
**Refinement**			
Initial model used (PDB code)	8I48	8WCR	8WCR
Model composition			
Non-hydrogen atoms	13,535	13,795	13,370
Protein residues	1,560	1,560	1,560
Ligands	30	40	30
CC mask	0.86	0.89	0.91
B factor (Å^2)			
Protein residues	106.13	88.44	63.54
Ligands	90.33	116.15	73.68
R.M.S. deviations			
Bond lengths (Å)	0.002	0.005	0.004
Bond angles (°)	0.537	0.594	0.557
Validation			
MolProbity score	1.43	1.57	1.27
Clashscore	4.42	3.82	2.55
Poor rotamers (%)	0.43	2.00	1.51
Ramachandran plot			
Favored (%)	96.65	97.03	97.55
Allowed (%)	3.35	2.97	2.45
Disallowed (%)	0	0	0

Supplementary Table 3. The Q-scores for residue 116 on the Pro-loop and residues 240-251 on the M2-M3 loop, calculated for all structures resolved at pH 4.0 and GLIC-F116A (pH 2.5). The reference expected Q scores were calculated by the reported map resolution using the Gold-Standard FSC method. For maps and models with C5 symmetry, only the Q-scores of chain A are listed. For maps and models with C1 symmetry, the Q-scores of residues on all chains are listed.

Model	F116	I240	L241	V242	E243	T244	N245	L246	P247	K248	T249	P250	Y251	Expected Q score
9LAG	0.73	0.64	0.66	0.61	0.57	0.56	0.63	0.52	0.51	0.65	0.44	0.66	0.65	0.59
9LAI-A	0.70	0.71	0.69	0.66	0.66	0.66	0.66	0.62	0.62	0.61	0.62	0.72	0.65	0.57
9LAI-B	0.72	0.71	0.68	0.64	0.66	0.71	0.62	0.62	0.60	0.66	0.55	0.66	0.66	
9LAI-C	0.75	0.70	0.69	0.61	0.62	0.68	0.66	0.56	0.59	0.61	0.50	0.59	0.64	
9LAI-D	0.73	0.72	0.62	0.64	0.65	0.56	0.62	0.57	0.56	0.58	0.37	0.66	0.62	
9LAI-E	0.76	0.62	0.67	0.56	0.53	0.68	0.50	0.53	0.71	0.67	0.60	0.70	0.71	
9LAJ-A	0.67	0.67	0.66	0.64	0.62	0.60	0.62	0.64	0.62	0.63	0.67	0.70	0.65	0.56
9LAJ-B	0.74	0.70	0.66	0.60	0.64	0.62	0.58	0.57	0.65	0.68	0.51	0.71	0.67	
9LAJ-C	0.73	0.68	0.66	0.66	0.62	0.42	0.55	0.52	0.60	0.62	0.38	0.59	0.58	
9LAJ-D	0.73	0.59	0.50	0.69	0.62	0.37	0.63	0.66	0.65	0.66	0.66	0.74	0.74	
9LAJ-E	0.74	0.48	0.39	0.48	0.35	0.39	0.63	0.49	0.64	0.52	0.64	0.71	0.72	
9LAK-A	0.70	0.66	0.67	0.66	0.63	0.68	0.64	0.52	0.61	0.63	0.64	0.70	0.64	0.55
9LAK-B	0.74	0.73	0.61	0.68	0.44	0.61	0.64	0.39	0.61	0.35	0.47	0.59	0.56	
9LAK-C	0.74	0.66	0.57	0.64	0.53	0.18	0.62	0.60	0.70	0.72	0.69	0.72	0.75	
9LAK-D	0.72	0.69	0.58	0.65	0.52	0.43	0.59	0.69	0.66	0.65	0.69	0.72	0.76	
9LAK-E	0.75	0.60	0.36	0.50	0.40	0.34	0.54	0.56	0.62	0.52	0.60	0.70	0.71	
9LB9-A	0.74	0.65	0.56	0.62	0.54	0.59	0.65	0.54	0.57	0.27	0.53	0.70	0.53	0.56
9LB9-B	0.76	0.61	0.62	0.66	0.64	0.36	0.74	0.67	0.72	0.72	0.69	0.77	0.76	
9LB9-C	0.74	0.71	0.61	0.64	0.66	0.27	0.62	0.67	0.74	0.68	0.73	0.80	0.79	
9LB9-D	0.75	0.70	0.56	0.65	0.55	0.32	0.62	0.70	0.72	0.64	0.68	0.79	0.74	
9LB9-E	0.77	0.66	0.50	0.63	0.63	0.45	0.60	0.63	0.68	0.65	0.70	0.74	0.74	
9LBA	0.77	0.70	0.64	0.66	0.65	0.29	0.66	0.66	0.72	0.70	0.75	0.79	0.78	0.61
9LBB-A	0.65	0.64	0.62	0.65	0.64	0.65	0.66	0.62	0.56	0.64	0.60	0.71	0.60	0.50
9LBB-B	0.69	0.67	0.57	0.63	0.64	0.41	0.58	0.48	0.45	0.27	0.53	0.63	0.62	

9LBB-C	0.73	0.56	0.52	0.53	0.46	0.62	0.47	0.49	0.69	0.58	0.62	0.65	0.69	
9LBB-D	0.65	0.68	0.56	0.57	0.50	0.55	0.59	0.48	0.46	0.47	0.46	0.63	0.59	
9LBB-E	0.72	0.53	0.55	0.53	0.62	0.61	0.42	0.44	0.64	0.55	0.62	0.63	0.64	
9LBC-A	0.70	0.65	0.52	0.63	0.59	0.63	0.67	0.59	0.54	0.62	0.55	0.52	0.49	0.51
9LBC-B	0.75	0.54	0.50	0.61	0.46	0.46	0.61	0.66	0.56	0.62	0.58	0.62	0.69	
9LBC-C	0.68	0.69	0.62	0.56	0.66	0.51	0.60	0.53	0.56	0.58	0.50	0.61	0.47	
9LBC-D	0.72	0.57	0.56	0.57	0.61	0.40	0.58	0.55	0.57	0.61	0.60	0.68	0.70	
9LBC-E	0.74	0.59	0.45	0.58	0.47	0.39	0.58	0.53	0.61	0.53	0.62	0.70	0.70	
9LBD	0.83	0.85	0.85	0.80	0.72	0.72	0.74	0.80	0.80	0.71	0.81	0.79	0.84	0.74
